# Marine Cyclic Peptides: Antimicrobial Activity and Synthetic Strategies

**DOI:** 10.3390/md20060397

**Published:** 2022-06-15

**Authors:** Ricardo Ribeiro, Eugénia Pinto, Carla Fernandes, Emília Sousa

**Affiliations:** 1 Laboratório de Química Orgânica e Farmacêutica, Departamento de Ciências Químicas, Faculdade de Farmácia, Universidade do Porto, Rua de Jorge Viterbo Ferreira 228, 4050-313 Porto, Portugal; ricardorc@live.com.pt (R.R.); esousa@ff.up.pt (E.S.); 2 Interdisciplinary Centre of Marine and Environmental Research (CIIMAR), Edifício do Terminal de Cruzeiros do Porto de Leixões, Avenida General Norton de Matos s/n, 4050-208 Matosinhos, Portugal; epinto@ff.up.pt; 3 Laboratório de Microbiologia, Departamento de Ciências Biológicas, Faculdade de Farmácia, Universidade do Porto, Rua de Jorge Viterbo Ferreira 228, 4050-313 Porto, Portugal

**Keywords:** marine peptides, cyclic peptides, cyclic depsipeptides, antimicrobial resistance, peptide synthesis

## Abstract

Oceans are a rich source of structurally unique bioactive compounds from the perspective of potential therapeutic agents. Marine peptides are a particularly interesting group of secondary metabolites because of their chemistry and wide range of biological activities. Among them, cyclic peptides exhibit a broad spectrum of antimicrobial activities, including against bacteria, protozoa, fungi, and viruses. Moreover, there are several examples of marine cyclic peptides revealing interesting antimicrobial activities against numerous drug-resistant bacteria and fungi, making these compounds a very promising resource in the search for novel antimicrobial agents to revert multidrug-resistance. This review summarizes 174 marine cyclic peptides with antibacterial, antifungal, antiparasitic, or antiviral properties. These natural products were categorized according to their sources—sponges, mollusks, crustaceans, crabs, marine bacteria, and fungi—and chemical structure—cyclic peptides and depsipeptides. The antimicrobial activities, including against drug-resistant microorganisms, unusual structural characteristics, and hits more advanced in (pre)clinical studies, are highlighted. Nocathiacins I–III (**91**–**93**), unnarmicins A (**114**) and C (**115**), sclerotides A (**160**) and B (**161**), and plitidepsin (**174**) can be highlighted considering not only their high antimicrobial potency in vitro, but also for their promising in vivo results. Marine cyclic peptides are also interesting models for molecular modifications and/or total synthesis to obtain more potent compounds, with improved properties and in higher quantity. Solid-phase Fmoc- and Boc-protection chemistry is the major synthetic strategy to obtain marine cyclic peptides with antimicrobial properties, and key examples are presented guiding microbiologist and medicinal chemists to the discovery of new antimicrobial drug candidates from marine sources.

## 1. Introduction

Despite great advances in the pharmaceutical and medicine fields, contagious diseases induced by bacteria, fungi, viruses, and protozoa are still a significant threat to public health, as evidenced by the SARS-Cov-2 pandemic [1]. Due to the emergence of new pathogenic agents, extensive resistance, and the lack of new drugs, contagious diseases affect both developed and developing countries [2,3]. 

The golden age of antibacterial agents began in the 1940–1960s, and many antibiotics dating from that period are still used in therapy today. Due to the high rate of antibiotics discovery, during this period, it was believed that infectious diseases would soon be controlled in the population [4]. A line of research on antimicrobial discovery and development was identified from many natural small-molecule products that were clinically proved to have antibacterial activity. Among these small molecules, penicillins, cephalosporins, macrolides, glycopeptides, tetracyclines and aminoglycosides stand out. On the other hand, another line of research was found from the structures of the chemical dye industry, leading to the discovery of aromatic sulfa compounds with antibiotic activity [5]. In 1960, the fluoroquinolones emerged, which are the second example of synthetic antibiotics used in therapy. Later, in the 2000s, the first generation of oxazolidinone linezolid, a synthetic derivative structurally different from the previous ones, was approved in the USA [6]. In addition, new generations of cephalosporins, macrolides, fluoroquinolones, and tetracyclines appeared with significant therapeutic use. It is important to highlight that the development of new synthetic methodologies has allowed the synthesis of pentacyclines, derived from tetracyclines, which may be considered as a fourth generation of this class of antibiotics [7].

Regarding the treatment of systemic mycoses, such as candidiasis, aspergillosis, and cryptococcosis, antifungals can be organized into four classes—polyenes, azoles, flucytosine, and echinocandins—in which they are distinguished by the mode of formulation, bioavailability, pharmacological interactions, adverse effects, and mechanism of action [8,9]. Although commensals in humans, *Candida* species are a cause of various infections in susceptible patients, including elderly, hospitalized, and immunosuppressed patients. Invasive *Candida* infection is one of the most common fungal infections globally [10]. Less common, but responsible for greater treatment concerns, are systemic infections caused by fungi of the genera *Aspergillus*, *Fusarium* and *Scedosporium*, considering their susceptibility profile to the available antifungals [11,12].

Over the course of human civilization, viral infections have caused millions of human casualties worldwide, driving the development of antiviral drugs in a pressing need [13]. As of April 2016, antiviral drugs have been approved to treat nine human infectious diseases: hepatitis B, hepatitis C, and infections caused by human immunodeficiency virus (HIV), human cytomegalovirus, herpes simplex virus (HSV), human papillomavirus, respiratory syncytial virus, varicella-zoster virus, and influenza virus [14,15]. Nevertheless, there is still no antiviral drug or vaccine for over 200 human viruses afflicting populations worldwide [14]. In addition, the current pneumonia outbreak caused by the severe acute respiratory syndrome coronavirus 2 (SARS-CoV-2) was declared a pandemic by the World Health Organization on 11 March 2020 [16].

Parasitic diseases are a critical health concern with a profound impact on the global human population [17]. It was found that protozoans, such as *Trypanosoma cruzi*, *Leishmania mexicana*, *Plasmodium falciparum*, *Giardia intestinalis*, and *Trichomonas vaginalis*, are the major disease-causing parasites. The spread of infectious diseases is especially prevalent in underdeveloped countries characterized by tropical or temperate climate, as well as poor sanitary and hygienic conditions [18,19,20]. Parasite infections are the cause of 500 million deaths worldwide [21,22], and although there are drugs to fight parasite infections, these have drawbacks such as toxicity and the emergence of resistance [23,24].

Antimicrobial resistance (AMR) is another significant threat to public health systems all over the world [25]. Infection caused by microorganisms resistant to antimicrobial drugs leads to serious illnesses and elevated costs associated with more expensive antibiotics (when infections become resistant to first-line antimicrobials, treatment has to be switched to second- or third-line drugs, which are nearly always more expensive), specialized equipment, longer hospital stay, and isolation procedures for the patients. Societal costs include loss of productivity and death [25,26]. Every year, more than 2 million North Americans acquire infectious diseases associated with antibiotic-resistant microorganisms, resulting in 23,000 deaths [27]. In Europe, nearly 700 thousand cases of antibiotic-resistant infections develop directly into over 33,000 deaths yearly [28]. Despite a 36% increase in human use of antibiotics from 2000 to 2010 [29], approximately 20% of deaths worldwide are related to infectious diseases today [30]. Statistical models predicted that there were an estimated 4.95 million deaths associated with AMR in 2019, including 1.27 million deaths associated with bacterial AMR [3]. The six leading pathogens associated with AMR in 2019 (*Escherichia coli*, followed by *Staphylococcus aureus*, *Klebsiella pneumoniae*, *Streptococcus pneumoniae*, *Acinetobacter baumannii*, and *Pseudomonas aeruginosa*) were responsible for 3.57 million deaths [3].

**Figure 1:**
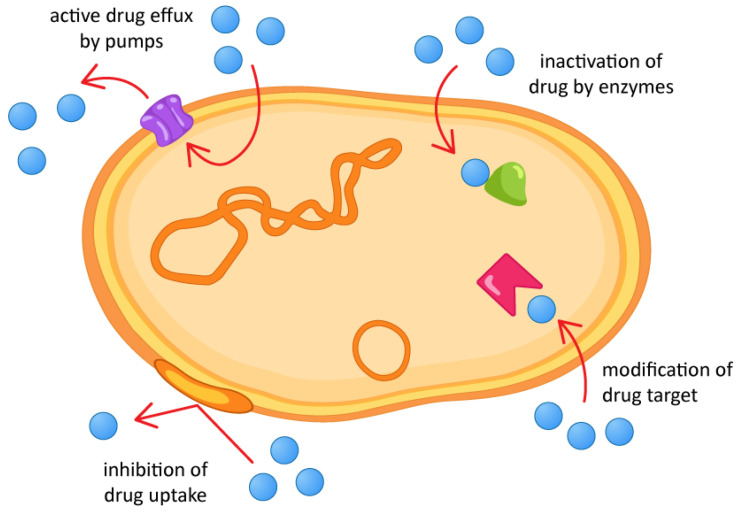
Antimicrobial resistance mechanisms.

AMR can occur through several mechanisms, whether intrinsic or acquired. Intrinsic resistance occurs naturally, as part of a microbial evolution process. Acquired resistance, on the other hand, is a consequence of the indiscriminate use of antimicrobials, and genetic mutations may occur, originating resistance genes that can be transferred between microbial species. AMR mechanisms fall into four main categories [31], as shown in Figure 1.

Ocean water covers about 70% of the Earth’s surface and contains several potential bioactive compounds to be discovered. Marine organisms have been considered to be a promising source of numerous nutraceutical and pharmaceutical compounds [32,33]. Over the last few decades, new marine-derived compounds have been considered not only as lead compounds for drug discovery, but also as bioactive agents in pharmaceutical research, possessing antifungal, antibacterial, cytotoxic and anti-inflammatory properties, among others [34,35,36,37].

Specific chemical and physical properties, such as water salt concentrations, pressure, temperature (including extreme), light penetration, ocean currents, oxygen concentrations, and radiation exposure characterize different underwater habitats (environment) of marine organisms [38,39]. Due to this extreme environment, marine organisms are forced to produce a chemical diversity of bioactive compounds that are considered essential for the discovery and development of new agents for the treatment and prevention of various fungal, bacterial, viral, and protozoal infections [35,40,41,42,43,44,45].

In particular, several peptides have been isolated from marine sources and demonstrated to be promising drug candidates based on their significance of the bioregulatory role and unique molecular mechanisms of action [46,47]. Our group also described the isolation and stereochemical analysis of marine peptides [48,49]. Compared to small-molecule drugs, peptides are highly selective and efficacious and, at the same time, relatively safe and well tolerated [50]. The high degree of selectivity in their interactions is the result of millions of years of evolutionary selection for complementary shapes and sizes from among a large array of structural and functional diversity [47]. 

Despite the applicability of peptides and proteins in medicine, it has been limited by their high manufacturing costs, the low bioactivity of peptides when administered orally, and their low membrane permeability, low systemic stability, and high clearance rates [51]. The low stability under physiological conditions is one of the main obstacles to the therapeutic use of linear peptides, as they easily lose their biological activity because they are rapidly cleaved by enzymes in vivo [52,53]. To overcome this obstacle, diverse peptide modifications have been proposed [54,55,56,57]. Linear peptide cyclization has recently been considered one of the most promising approaches, due to several advantages in surpassing both pharmacokinetic and pharmacodynamic limitations. A cyclic structure reduces the conformational freedom for each constituent within the cycle and forces the molecule into a more rigid secondary structure [58]. The increase in rigidity is an advantage that is translated into a decrease in the entropic term of Gibbs energy, improving binding affinities higher than some natural ligands to a biotarget [59,60,61,62]. Common motifs in the formation of proteins and polypeptides, *β*-turn, are other advantages of cyclization, as it is believed that this improves binding affinity [63,64,65]. Cyclization also allows the elimination of charged terminals at the ends of the structure in cyclic peptides, which may increase membrane permeability [66], although the peptide cross of the membrane does not improve just because the peptide is cyclized, but due to its structural features [67]. Another advantage of cyclization is becoming less prone to hydrolysis, as it decreases the exposure of the amino and carboxyl termini to exopeptidases [68], decreasing off-target side effects [69], thus leading to substantially enhanced metabolic stability and specificity [70,71,72,73]. Furthermore, in terms of chemical synthesis, cyclic peptides are significantly smaller when compared to proteins, and therefore, lower manufacturing costs are needed [74]. Actually, cyclic peptides are polypeptide chains that contain a circular sequence of bonds, which can be formed through a bond between the amino and carboxyl termini of the peptide with an amide bond, or other chemically stable bonds such as lactone, ether, thioether, and disulfide, among others. The formation of the amide bond between the amino and carboxylic terminals results in the formation of a head-to-tail (or *N*-to-*C*) cyclic peptide. Many cyclic peptides with this kind of formation (*N*-to-*C*) exist in nature [75,76,77,78,79].

Depending on lipophilicity, the type of bonds between amino acids, and the number of amino acids, cyclic peptides can have different classifications, as either cyclic lipopeptides, cyclic glycopeptides, or cyclic depsipeptides. Cyclic lipopeptides are cyclic peptides acylated by a lipid, usually a fatty acid (FA) side chain. These compounds are produced only in bacteria and fungi of various habitats during cultivation on carbon and nitrogen sources [80]. Cyclic glycopeptides contain carbohydrate moieties covalently attached to the side chains of amino acid residues [81]. Cyclic depsipeptides are cyclic peptides in which amide groups are replaced by corresponding lactone bonds due to the presence of a hydroxy-carboxylic acid in the peptide structure by cyclization to the hydroxyl of serine or threonine side chains [82]. Modification of the amide bond to an ester increases the lipophilicity that may subsequently improve cell permeability. Depsipeptides have also been used to demonstrate the importance of the hydrogen bonds that are formed by amide bonds in natural peptides [79]. These peptides sometimes display additional chemical modifications, including unusual amino acid residues in their structures. Cyclic depsipeptides contain at least one ring formed only through peptide or ester links—derived from hydroxy carboxylic acids [83]. Cyclic peptides and cyclic depsipeptides can be named as cyclic tri-, tetra-, penta-, hexa-, hepta-, octa-, nona-, deca-, unde-, dodeca, and tri-decapeptides, depending on the number of amino acids present [84].

Depsipeptides are also called peptolides or nonribosomal peptides (NRP), being biosynthesized by non-ribosomal peptide synthetases (NRPS) in combination with either polyketide synthase (PKS), or FA synthase enzyme systems, and are often found in marine organisms such as bacteria, tunicates, mollusks, and sponges, among others [76,77,78]. NRPS are multifunctional proteins that synthesize peptide natural products independent of the mRNA ribosomal machinery and employ a modular architecture wherein each module is responsible for the incorporation of one amino acid into the final peptide product [85,86]. NRP can be linear, cyclic, or branched peptides, and usually contain fewer than 20 amino acid residues and are often modified by chemical processes such as acylation, glycosylation, and others. Each module functions as a building block responsible for the incorporation and modification of an amino acid, especially D-amino acids, so that the order and quantity of modules in an NRPS determine the amino acid sequence of the synthesized peptide [87]. 

**Figure 2:**
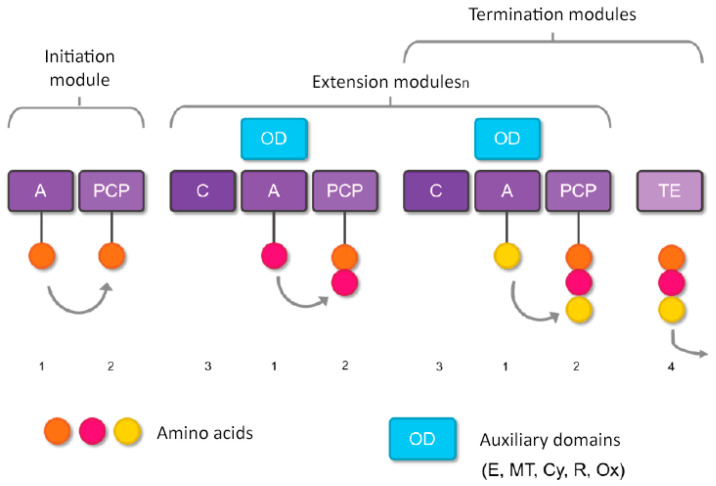
Basic steps of nonribosomal peptide synthesis. (1) Domain A selects the amino acid to be incorporated and transfers it to the PCP domain (2), where a thioester bond is formed. (3) Domain C forms the peptide bond between the amino acid present in the PCP domain of the same module and the intermediate peptidyl linked to the PCP domain of the previous module (that is, it catalyzes the link between amino acids of adjacent modules). (4) If no additional domains are present that modify the molecule during formation, the TE domain releases the formed peptide. However, if additional domains are present (such as E, MT, Cy, or Ox), the molecule is modified before being released by the TE domain.

The section of the NRPS enzyme that specifically incorporates an amino acid into the peptide chain is defined as a module, and the modules, in turn, can be divided into domains, which catalyze the individual steps of non-ribosomal peptide synthesis. Each standard elongation module consists of three domains: a condensation domain (C), an adenylation domain (A), and a peptidyl transporter protein (PCP), organized as C-A-PCP [87] (Figure 2). The PCP domain is also frequently referred to as the T domain, and the holoform of this domain is a substrate for thioester (“thiolation”) formation [88,89,90,91,92]. In addition to these main domains, there are others involved in modifying an NRP: the E domain (molecule epimerization); the Cy domain (cyclization of the forming molecule); the MT domain (methylation reactions); the R domain (reduction reactions), and the Ox domain (oxidation reactions) [93].

The terminating domain, the thioesterase (TE) domain, normally releases the peptide by hydrolysis or cyclization [94], while reductase (R) domains catalyze release by converting the thioester link to an aldehyde [95], or alcohol [96], specialized C domains [97], catalyze cyclization [98], and amide bond formation through small molecule acceptance [97,99,100,101].

In recent decades, several fundamental reviews have assembled the main achievements related to chemical synthesis [102,103,104,105], biosynthesis [88,89,90,91,92,106], chemical classification [47,82], absolute configuration measuring [107], and applications [75,108,109] of marine cyclic peptides. In addition, there are some reviews related to marine-derived compounds with antifungal activity [110] or focusing on antimicrobial drug resistance concern with marine natural products [111], including marine cyclic peptides. In this review, a comprehensive compilation of marine cyclic peptides with relevant antimicrobial activity described throughout the literature is the main relevance and novelty of this review compared to others. This review focuses on cyclic peptides from marine sources which demonstrated potential antimicrobial activity against several microorganisms (in vitro and some in vivo studies), including drug-resistant fungal and bacterial strains. Peptides containing unusual structural characteristics or subjected to advanced investigations as far as possible, supported by comparison applying conventional drug treatment, are emphasized. In addition, relevant synthetic approaches for the total synthesis of the described peptides are highlighted.

## 2. Marine Cyclic Peptides with Antimicrobial Activities

In this section, marine cyclic peptides organized by producer marine species and scaffold are represented in alphabetical order. The antimicrobial activities of compounds **1**–**174**, from several marine sources (sponges, bacteria, fungi, tunicate, other as ascidian), demonstrated potential antimicrobial activity (in vitro and some in vivo) against several microorganisms, including drug-resistant fungal and bacterial strains, antiparasitic, and antiviral effects. The total or semi-synthesis of marine cyclic peptides, analogues, and/or fragments are also reported and summarized in Table 1, Table 2, Table 3, Table 4 and Table 5.

### 2.1. Sponge-Produced Cyclic Peptides

**Figure 3:**
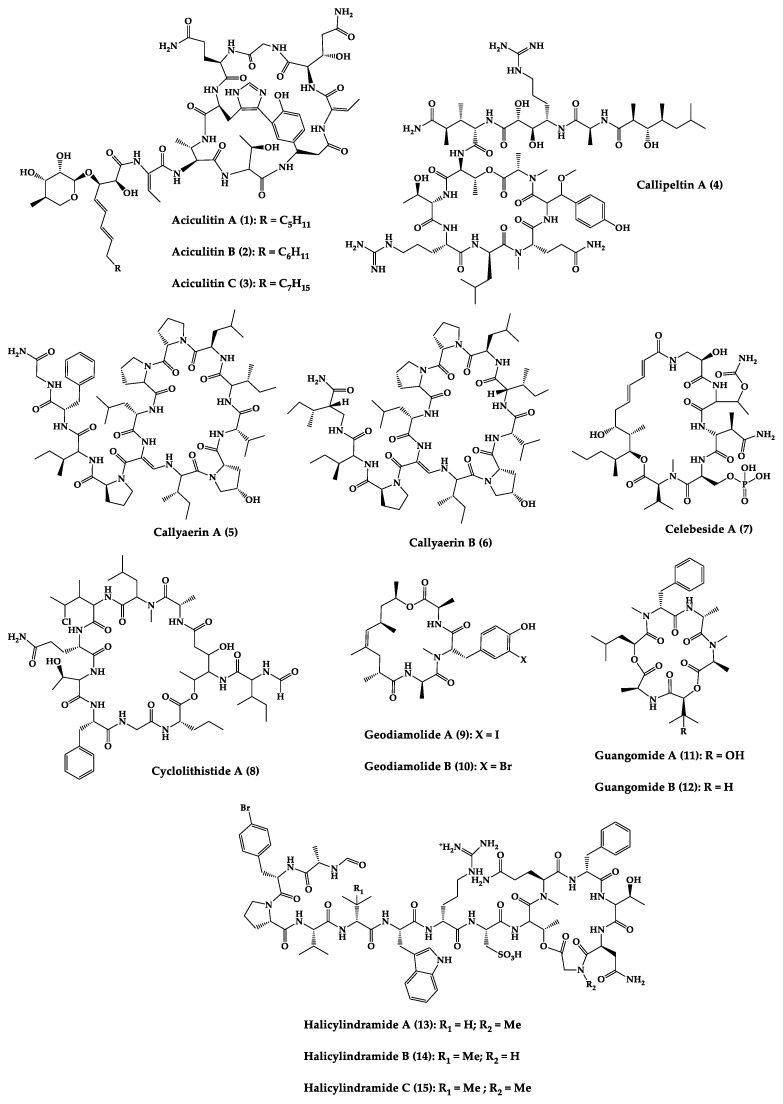

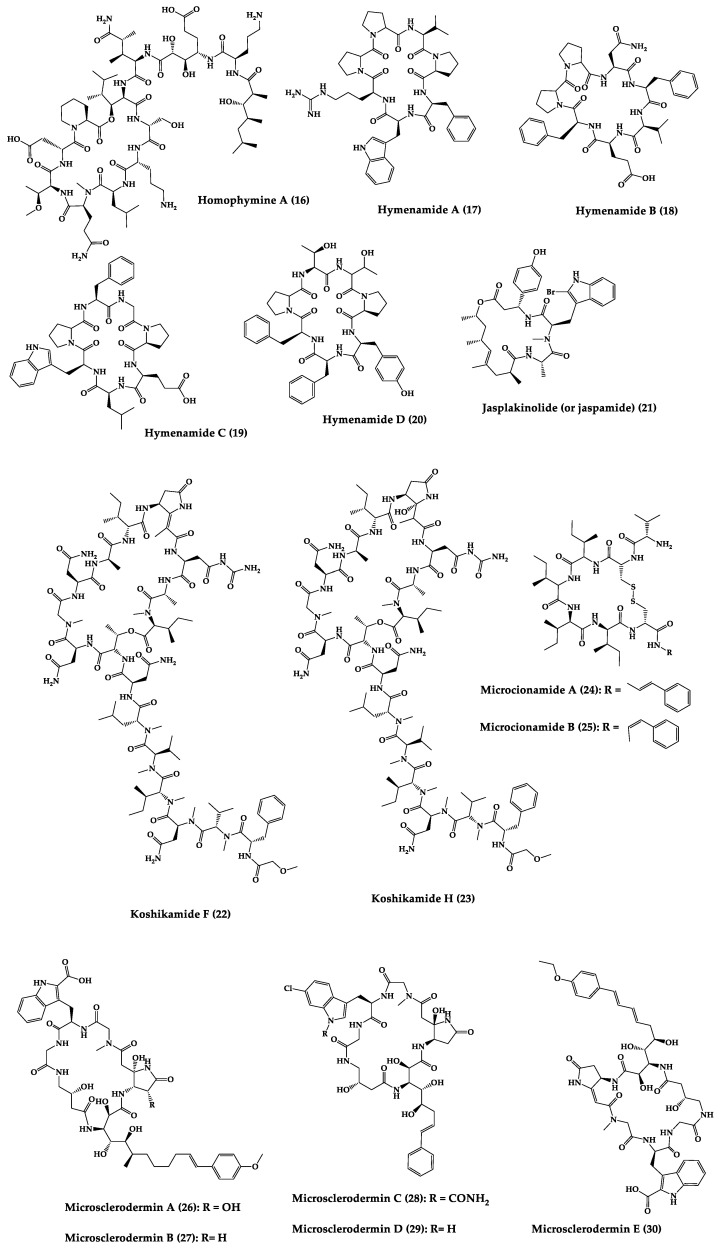

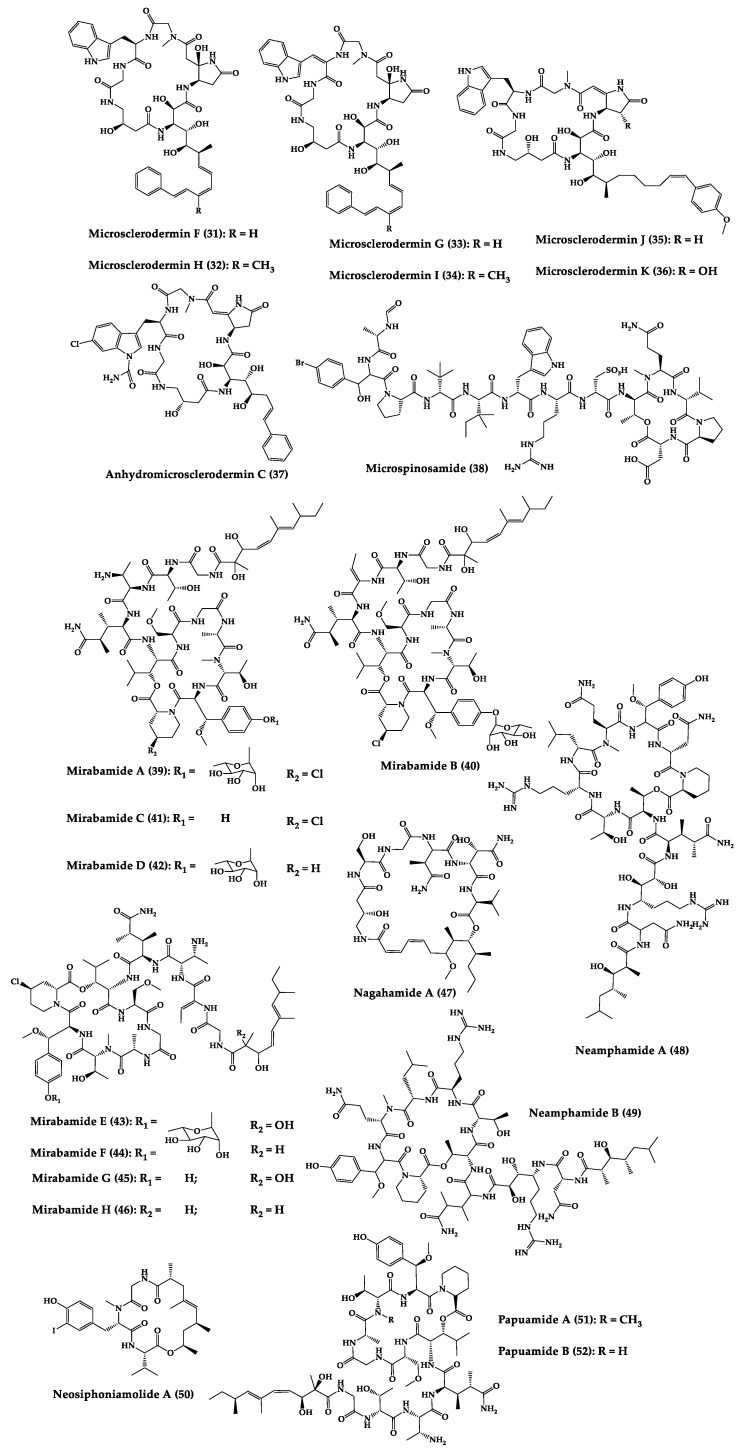

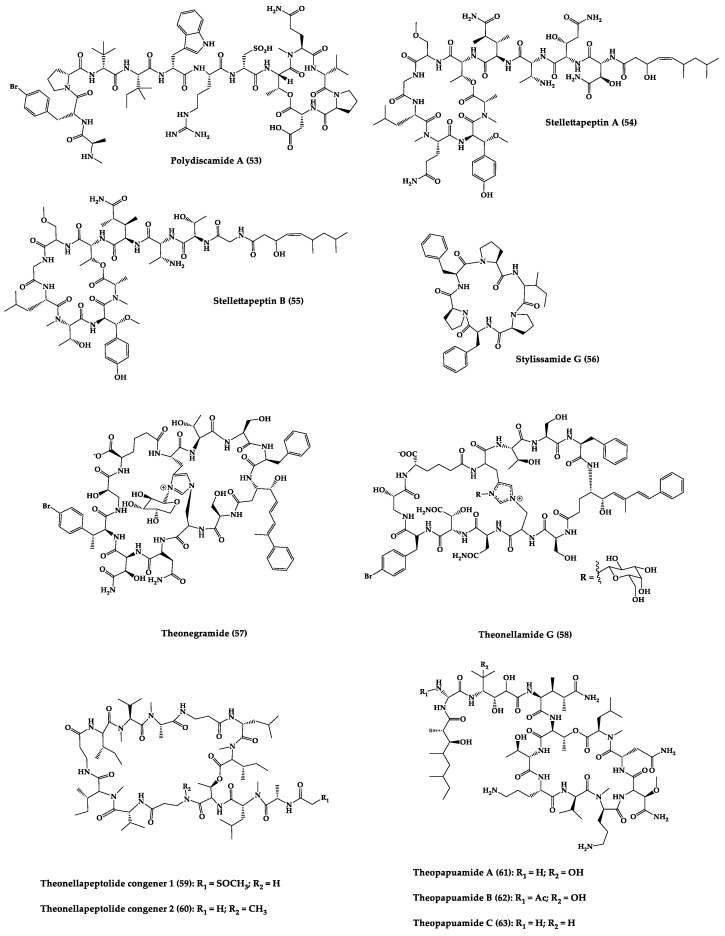
Chemical structures of cyclic peptides from sponges (**1**–**63**).

Sponges are diversified organisms distributed extensively on shores and deep in the ocean [112]. In terms of chemical diversity, an exceptionally prolific group of sponges include the lithistid sponges, the prominence of which may be due to the biosynthetic capacity of the microorganisms that host them [113,114,115]. The metabolites of lithistid sponges, which include the genera *Theonella*, *Discodermia*, *Aciculites*, *Microscleroderma*, and *Callipelta*, are among the most diverse found in any order of sponges and have often been attributed to symbiotic microorganisms (such as proteobacteria “*Candidatus* Entotheonella palauensis”) [114], that contains four distinct cell populations: sponge cells, unicellular heterotrophic bacteria, unicellular cyanobacteria, and filamentous heterotrophic bacteria. In particular, many cyclic peptides and peptide lactones have been reported to be able to be obtained from *Theonella* sponges, and their structural features, including unusual amino acids or D-amino acids, suggest that they perhaps originated from symbiotic microorganisms [116,117]. In this section, 63 cyclic peptides isolated from sponges (**1**–**63**) are described (Table 1 and Figure 3). Among these, 14 cyclic peptides have been found to have antibacterial activities, as well as 19 with antifungal, one with parasitic, and 22 with antiviral activities. The most relevant antimicrobial cyclic peptides are highlighted due to their unusual structural characteristics or advanced investigations, potency, and in vivo experiments. 

Halicylindramides A–C (**13**–**15**) were isolated from the Japanese marine sponge *Halichondria cylindruta* and are cyclic tetradecapeptides with the *N*-terminus blocked by a formyl group and the *C*-terminus lactonized with a threonine residue. Compounds **13**–**15** demonstrated in vitro antifungal activity against *Mortierella ramanniana* at 7.5 µg/disk. Interestingly, it was found that the macrocyclic structure of compounds **13**–**15** is essential for their cytotoxic and antifungal activities [118].

An anti-HIV undecadepsipeptide, designated homophymine A (**16**), isolated from a New Caledonian collection of the marine sponge *Homophymia* sp, contains 11 amino acid residues and an amide-linked 3-hydroxy-2,4,6-trimethyloctanoic acid moiety. This undecadepsipeptide exhibited in vitro cytoprotective activity against HIV-1 infection, demonstrating a half-maximal inhibitory concentration (IC_50_) of 75 nM [119] in a cell-based XTT assay.

Jasplakinolide (**21**), also named jaspamide, is a 19-membered macrocyclic depsipeptide isolated from the soft-bodied sponge *Jaspis* species collected off the shore of the island of Benga, Fiji, and shows selective in vitro antimicrobial activity with a minimum inhibitory concentration (MIC) greater than 25 µg/mL against *Candida albicans*. The in vivo topical activity of a 2% solution of **21** against a Candida vaginal infection in mice was similar to that of miconazole nitrate (MIC = 6.2 µg/mL) [120,121]. This macrocyclic (**21**) exhibited insecticidal activity against *Heliothis virescens*, with a lethal concentration in 50% of the population (LC_50_) of 4 ppm. It was also toxic to the nematode *Nippo*-*Strongylus brasiliensis* with a lethal dose in 50% of the population (LD_50_) of 1 µg/mL [122].

Koshikamides F (**22**) and H (**23**) are 17-residue cyclic heptadecadepsipeptides containing a 10-residue macrolactone, isolated from deep-water Palauan collections of *T. swinhoei* and *T. cupola*. Linear koshikamides (data not shown) failed to inhibit HIV entry, while the cyclic peptides **22** and **23** inhibited HIV entry with IC_50_ values of 2.3 and 5.5 μM, respectively. Thus, the presence of the exocyclic olefin and its associated conformation appear to enhance activity relative to the hydroxypyrrolidone version, suggesting the ten-residue lactone ring is important for inhibition of HIV-1 entry. Lastly, the slightly more potent anti-HIV activity of **22** and **23** may be due to the distinct conformation of the macrolactone brought about by the presence of the unsaturated pyrrolidinone residue 2-(3-amino-5-oxopyrrolidin-2-ylidene)propanoic acid [123].

Microspinosamide (**38**), a cyclic tridecadepsipeptide, incorporates numerous uncommon amino acids, and it was the first naturally occurring peptide described to contain a *β*-hydroxy-*p*-bromophenylalanine residue. Microspinosamide (**38**) inhibited the cytopathic effect of HIV-1 infection in an XTT-based in vitro assay with an effective concentration in 50% population (EC_50_) value of approximately 0.2 µg/mL [124].

Four cyclic glycodepsipeptides have been isolated from the marine sponge *Siliquariaspongia mirabilis*, namely mirabamides A–D (**39**–**42**), which contain 4-chlorohomoproline in **39**, **40** and **41**, and an unusual glycosylated amino acid, *β*-methoxytyrosine 4′-*O*-R-L-rhamnopyranoside, along with a *N*-terminal aliphatic hydroxy acid. Mirabamide A (**39**) was demonstrated in vitro to inhibit HIV-1 in neutralization and fusion assays, with IC_50_ values between 40 nM and 140 nM, while mirabamides C (**41**) and D (**42**) presented IC_50_ values of 140 nM–1.3 µM and 190 nM–3.9 µM, respectively. These results indicate that these peptides can act in the early stages of HIV-1 entry. Additionally, mirabamides E–H (**43**–**46**), which were isolated from the sponge *Stelletta clavosa*, collected from the Torres Strait, demonstrated in vitro HIV-1 inhibition in a neutralization assay, with IC_50_ values of 121 nM, 62 nM, 68 nM, and 41 nM, respectively. Some interesting structure–activity relationships (SAR) emerged by comparing the HIV inhibitory activities of mirabamides E–H (**43**–**46**) with those previously determined for mirabamides A–D (**39**–**42**) [125]. The primary feature that distinguishes compounds **43**–**46** from **39**–**42** is the presence of 2-amino-2-butenoic acid rather than threonine, and this change was found to improve activity (evidenced by the 2-fold increase in potency of **45** compared to **41**). In general, increasing hydrophobicity in the side chain, but not including 2,3-diaminobutanoic acid (polar headgroup), improved potency. A potential model that may account for this tendency involves the inclusion of the hydrophobic tail into the plasma membrane by the presence of the polar headgroup (such as 2-amino-2-butenoic acid) [126]. The role of the rhamnose residue in potency is less clear. For **43**–**46**, the absence of rhamnose is correlated with improved activity (∼2-fold) in neutralization assays, whereas the absence of rhamnose was associated with an increase and a decrease in activity for mirabamide C (**41**) vs. A (**39**) and mirabamide D (**42**) vs. papuamide A (**51**), respectively, in HIV-1 envelope-mediated fusion assays [125].

Another interesting example is neamphamide A (**48**), an HIV-inhibitory cyclic undecadepsipeptide, isolated from a Papua New Guinean collection of the marine sponge *Neamphius huxleyi*, containing 11 amino acid residues and an amide-linked 3-hydroxy-2,4,6-trimethylheptanoic acid moiety. The anti-HIV activity of **48** was evaluated in an XTT-based cell viability assay using the human T-cell line CEMSS infected with HIV-1_RF_ [127]. After a 6-day incubation period, compound **48** effectively inhibited the cytopathic effect of HIV-1 infection with an EC_50_ = 28 nM [128].

Neamphamide B (**49**), a cyclic undecadepsipeptide, isolated from a marine sponge of *Neamphius* sp. collected at Okinawa, Japan, consists of uncommon amino acid residues (11 amino acid residues and an amide-linked 3-hydroxy-2,4,6-trimethylheptanoic acid moiety) and *N*-terminal aliphatic hydroxyl acid. The peptide **49** showed potent anti-mycobacterial activity against *Mycobacterium smegmatis* under standard aerobic growth conditions, as well as dormancy inducing hypoxic conditions with MIC of 1.56 µg/mL. Compound **49** was also effective against *Mycobacterium bovis* with MIC in the ranging of 6.25–12.5 µg/mL [129].

Papuamide A (**51**) and B (**52**) are cyclic depsipeptides isolated from bacteria symbiosis sponges *Theonella mirabilis* and *Theonella swinhoei* that exhibit a concentration-dependent increase in human T-lymphoblastoid cellular viability, indicating an inhibition of productive infection relative to control cultures, with an EC_50_ of 3.6 ng/mL. The HIV-inhibitory and cytotoxic activities of **52** in the same assay were virtually identical to those observed for **51** [130]. 

Theonellamide G (**58**) is a bicyclic glycodepsipeptide collected from bacteria symbiosis red sea sponge *Theonella swinhoei* that showed in vitro antifungal activity towards wild and amphotericin B-resistant strains of *C*. *albicans* with IC_50_ of 4.49 μM and 2.0 μM, respectively, compared to 1.48 μM for the positive antifungal control amphotericin-B against the wild type [131].

Theopapuamide A–C (**61**–**63**) are cyclic undecadepsipeptides isolated from bacteria symbiosis sponges *Theonella swinhoei* and *Siliquariaspongia mirabilis*, which contain several unusual amino acid residues, where the occurrence of *α*-methoxyasparagine and 4-amino-5-methyl-2,3,5-trihydroxyhexanoic acid is unprecedented in natural peptides. The compounds **61**–**63** inhibited the in vitro growth of wild-type and amphotericin B-resistant wild-type strain of *C*. *albicans* at loadings of 1–5 µg/disk, displaying zones of growth inhibition of 8 mm [132]. All theopapuamides, which lack a *β*-methyltyrosine residue, were inactive [125]; however, further studies demonstrated that theopapuamide B (**59**) was active in the neutralization assay [133] with an IC_50_ of 0.8 μg/mL, in an in vitro single-round HIV-1 infectivity assay against viruses pseudo-typed with HIV-1 SF162 envelope [133]. Ratnayake et al. [132] suggested that the *β*-methyltyrosine residue was critical for the anti-HIV activity. 

According to the overall results, some remarks can be inferred. The antiviral activity results reported for mirabamide A (**39**) and papuamide A (**51**), which both contain a *β*-methoxytyrosine, may be justified by the fact that this residue imparts a specific conformation required for binding to target protein involved in HIV-1 entry [125]. In the case of homophymine A (**16**), in which the *β*-methoxytyrosine is replaced by an *O*-methyl threonine (a smaller portion of moiety and more polar), the hypothesis that *β*-methoxytyrosine is essential for antiviral activity is ruled out [119]. 

**Table 1:** Antimicrobial cyclic peptides from marine sponges.

Compound	Structure	Source	Antimicrobial Activity	Synthesis	References
Aciculitins A-C (**1**–**3**)	Bicyclic octa-peptides	*Aciculites orientalis*	*C*. *albicans* (2.5 µg/disk, standard disk assay)	Semi-synthesis	[134,135]
Callipeltin A (**4**)	Cyclic deca-depsipeptide	*Callipelta* sp.	HIV-1 infection inhibition (CD_50_ = 0.29 µg/mL, ED_50_ = 0.01 µg/mL), *C*. *albicans* (100 µg/disk)	Total synthesis of analogues	[136,137,138,139,140]
Callyaerins A (**5**) and B (**6**)	Cyclic undeca-peptides	*Callyspongia aerizusa*	IC_90_: *M*. *tuberculosis* (2 μM and 5 μM, respectively), isoniazide (0.625 μM)	Total synthesis	[141,142]
Celebeside A (**7**)	Cyclic penta-depsipeptide	*Siliquaria-spongia mirabilis*	IC_50_: Neutralized HIV-1 (1.9 µg/mL)	-	[133]
Cyclolithistide A (**8**)	Cyclic deca-despipeptide	Bacteria symbiosis *Theonella swinhoei*	*C*. *albicans* (20 µg/disk)	-	[143]
Geodiamolides A (**9**) and B (**10**)	Cyclic depsipeptides	*Geodia* sp.	MIC: *C*. *albicans* (31.3 µg/mL)	Total synthesis	[144,145,146]
Guangomides A (**11**) and B (**12**)	Cyclic tetra-depsipeptides	Unidentifiable sponge derived fungus	MIC: *S*. *epidermidis* (100 µg/mL), *E*. *durans* (100 µg/mL)	-	[147]
Halicylindramides A-C (**13**–**15**)	Cyclic tetra-decapeptides	*Halichondria cylindruta*	*M*. *ramanniana* (7.5 µg/disk)	Total synthesis and analogues	[118,148,149]
Homophymine A (**16**)	Cyclic undeca-depsipeptide	*Homophymia* sp.	IC_50_: HIV-1 infection cytoprotective (75 nM)	Semi-synthesis	[119,150,151]
Hymenamides A (**17**), B (**18**), C (**19**), and E (**20**)	Cyclic hepta-peptides	*Hymeniacidon* sp.	MIC: *C*. *albicans* (33–66 µg/mL), *C*. *neoformans* (33–133 µg/mL)	Total synthesis and analogues	[152,153,154]
Jasplakinolide (or jaspamide) (**21**)	Cyclic depsipeptide	*Jaspis* sp.	*H*. *virescens* (LC_50_ = 4 ppm), *N*. *brasiliensis* (LD_50_ < 1 µg/mL), *C*. *albicans* (MIC > 25 µg/mL), in vivo murine vaginal *C*. *albicans* infection (2% jasplakinolide was equivalent in efficacy to administration of miconazole nitrate at 2%)	Total synthesis and analogues	[120,121,122,155,156,157]
Koshikamides F (**22**) and H (**23**)	Cyclic heptadeca-peptides	*Theonella swinhoei* and *T. cupola*	IC_50_: HIV-1 neutralization (2.3–5.5 µM)	-	[45,123]
Microcionamides A (**24**) and B (**25**)	Cyclic hexapeptides	*Clathria abietina*	MIC: *M*. *tuberculosis* (5.7 µM)	-	[158]
Microsclero-dermins A–K (**26**–**36**) and anhydromicros-clerodermin C (**37**)	Cyclic hexapeptides	Cyanobacteria simbiosis *Microsclero-derma herdmani* sp. and *Theonella* sp.	*C*. *albicans* (2.5–100 µg/disk, standard disk assay)	Total synthesis and analogues	[159,160,161,162,163]
Microspinosamide (**38**)	Cyclic trideca-depsipeptide	*Sidonops microspinosa*	EC_50_: HIV-1 infection inhibition (0.2 µg/mL)	Semi-synthesis	[124,164]
Mirabamides A–H (**39**–**46**)	Cyclic glyco-depsipeptides	*Siliquarias-pongia mirabilis* and *Stelletta clavosa*	IC_50_: neutralized and fusion HIV-1 (40 nM–3.9 µM), *B*. *subtilis*, *C*. *albicans* (1–5 µg/disk)	Semi-synthesis	[125,126,165]
Nagahamide A (**47**)	Cyclic hexa-depsipeptide	*Theonella swinhoei*	*E*. *coli* or *S*. *aureus* (50 µg/disk, inhibition zone 7 mm)	Semi-synthesis	[166,167]
Neamphamide A (**48**)	Cyclic undeca-depsipeptide	*Neamphius huxleyi*	EC_50_: HIV-1 infection cytoprotective (28 nM)	-	[128]
Neamphamide B (**49**)	Cyclic undeca-depsipeptide	*Neamphius* sp.	MIC: *M*. *smegmatis* (1.56 µg/mL), *M*. *bovis* (6.2–12.5 µg/mL)	-	[129]
Neosiphoniamolide A (**50**)	Cyclic tetra-depsipeptide	*Neosiphonia suprtes*	*P*. *oryzae* (IC_90_ = 5 ppm) *H*. *gramineum* (MIC ≤ 2 µg/mL)	-	[168]
Papuamides A (**51**) and B (**52**)	Cyclic depsipeptides	Bacteria symbiosis *Theonella mirabilis* and *Theonella swinhoei*	EC_50_: HIV-1 infection inhibition (1–74 ng/mL)	Total synthesis and analogues	[130,165,169,170,171,172]
Polydiscamide A (**53**)	Cyclic tridecapeptide	*Discodermia* sp.	MIC: *B*. *subtilis* (3.1 µg/mL)	Total synthesis and analogues	[173,174]
Stellettapeptins A (**54**) and B (**55**)	Cyclic undecadepsi-peptides	Microorganisms symbiosis *Stelletta* sp.	EC_50_: infection of human T-lymphoblastoid cells by HIV-1_RF_ (23 and 27 nM, respectively)	-	[175]
Stylissamide G (**56**)	Cyclic heptapeptide	*Stylissa caribica*	MIC: *M. audouinii*, *T. mentagrophytes*, *C. albicans* (6 μg/mL)	Total Synthesis	[176]
Theonegramide (**57**)	Bicyclic glycododecapeptide	Bacteria symbiosis *Theonella swinhoei*	*C*. *albicans* (10 µg/disk)	-	[177]
Theonellamide G (**58**)	Bicyclic glyco-depsipeptide	Bacteria symbiosis *Theonella swinhoei*	IC_50_: Wild and amphotericin B-resistant strains of *C*. *albicans* (2.0–4.49 μM), amphotericin-B (1.48 μM)	Semi-synthesis	[131,178]
Theonellapeptolide congeners 1 (**59**) and 2 (**60**)	Cyclic trideca-depsipeptides	*Theonella* sp.	MIC: *S*. *aureus* (8.0–16 µg/mL), *M*. *luteus* (8.0 µg/mL), *B*. *subtilis* (8.0–16 µg/mL), *M*. *smegmatis* (16–66 µg/mL), *T*. *mentagrophytes* (4.0–8.0 µg/mL), *A*. *niger* (8.0–66 µg/mL)	Total synthesis and analogues	[179,180]
Theopapuamide A-C (**61**–**63**)	Cyclic undeca-depsipeptides	Bacteria symbiosis *Theonella swinhoei* and *Siliquarias-pongia mirabilis*	Wild type and amphotericin B-resistant strains of *C*. *albicans* (1–5 µg/disk); in vitro HIV-1 infectivity assay IC_50_ = 0.8 μg/mL	-	[133]

CD_50_ (median convulsant); EC_50_ (effective concentration in 50% of population); ED_50_ (effective dose in 50% of population); HIV (human immunodeficiency virus); IC_50_ (half maximal inhibitory concentration); IC_90_ (maximum inhibitory concentration in 90% population); LD_50_ (lethal dose in 50% population); MIC (minimum inhibitory concentration). *Aspergillus niger* (*A*. *niger*); *Bacillus subtilis* (*B*. *subtilis*); *Candida albicans* (*C*. *albicans*); *Cryptococcus neoformans* (*C*. *neoformans*); *E*. *coli* (*Escherichia coli*)*; Enterococcus durans* (*E*. *durans*); *Heliothis virescens* (*H*. *virescens*); *Helminthosporium gramineum* (*H*. *gramineum*); *Micrococcus luteus* (*M*. *luteus*); *Microsporum audouinii* (*M. audouinii*); *Mortierella ramanniana* (*M*. *ramanniana*); *Mycobacterium* species (*M*. *bovis*, *M*. *smegmatis*, *M*. *tuberculosis*); *Nippo*-*Strongylus brasiliensis* (*N*. *brasiliensis*); *Piricularia oryzae* (*P*. *oryzae*); *Staphylococcus* species (*S*. *aureus*, *S*. *epidermidis*); *Trichophyton mentagrophytes* (*T*. *mentagrophytes*).

### 2.2. Bacteria-Produced Cyclic Peptides

**Figure 4:**
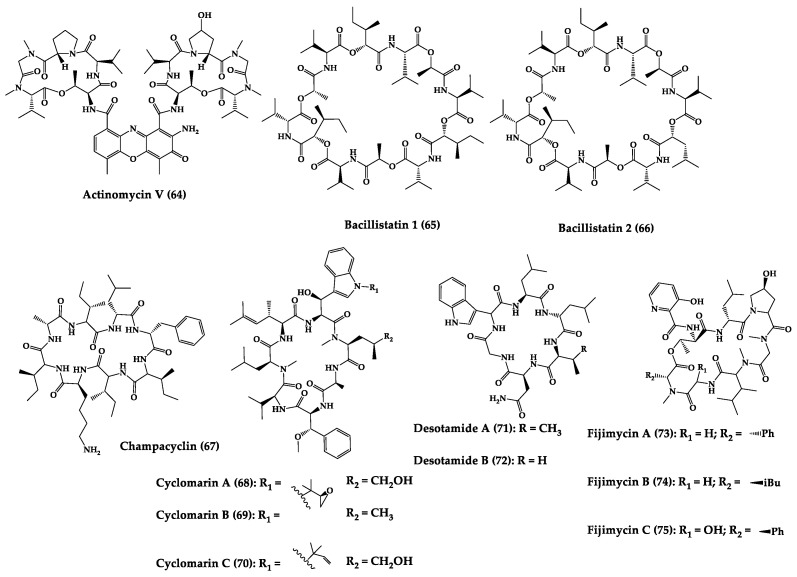

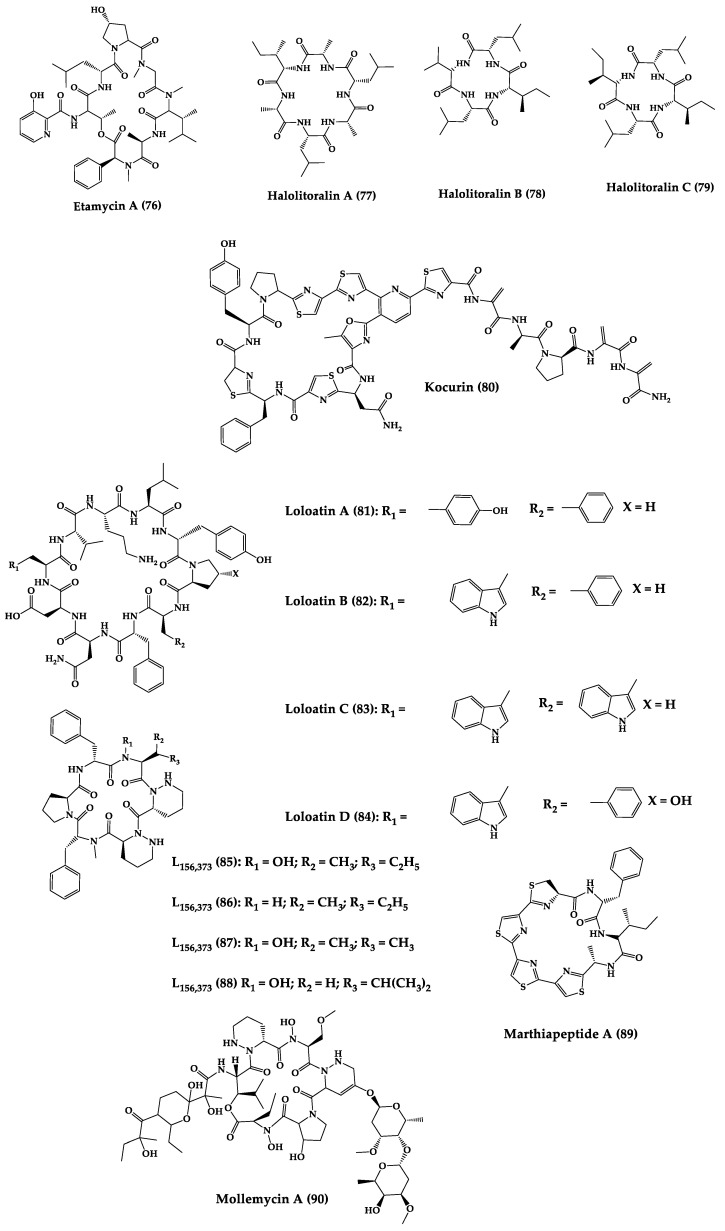

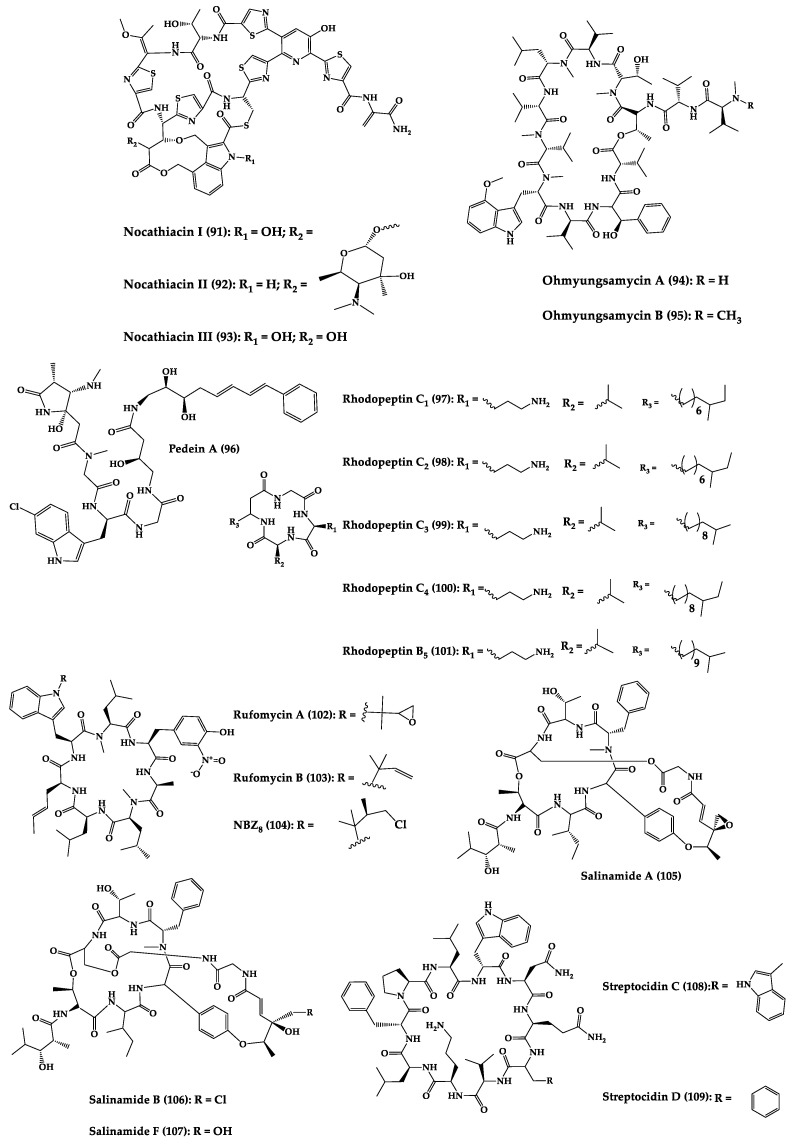

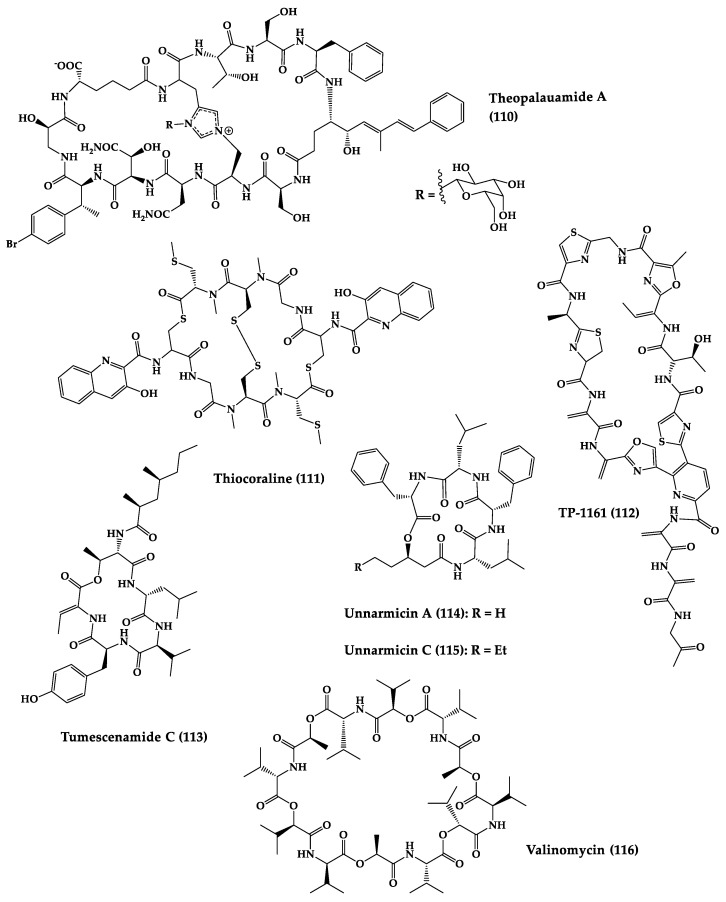
Chemical structures of cyclic peptides from bacteria (**64**–**116**).

A great variety of bacteria can be found in different marine habitats. Recent studies have shown that the main phyla of marine bacteria have a wide range of inhibitory activity against different types of microorganisms. Many antimicrobial substances active in a wide range of target organisms are produced by marine bacteria [181]. In this section, 53 cyclic peptides isolated from bacteria (**64**–**116**) have been reported (Figure 4 and Table 2). Among these, 38 cyclic peptides have been described with antibacterial activities, as well as 14 with antifungal, five with antiparasitic, and one with antiviral activities. The most relevant antimicrobial cyclic peptides are highlighted here due to their unusual structural characteristics or advanced investigations, potency, and in vivo experiments. 

Cyclomarins A–C (**68**–**70**) isolated from marine *Streptomyces* sp. are potent inhibitors of *Plasmodium falciparum*, whose biotarget was found to be the diadenosine triphosphate hydrolase [182]. Cyclomarin C (**70**) showed activity against multidrug-resistant Plasmodium falciparum strains (IC_50_ = 0.25 μM), as well as anti-tuberculosis activity (MIC of 0.1 μM) [183].

Four cyclic heptapeptides, with the assigned code of L-156,373 and three derivatives (**85**–**88**), were isolated from a culture of the marine *Streptomyces* sp. The heptapeptides investigated in this study showed significant activities against the pathogens *S*. *aureus*, MRSA, Bacillus Calmette-Guérin, and *B*. *subtilis*, with low MIC values of 0.025 to 1.25 μg/mL. The anti-*S*. *aureus* and anti-methicillin-resistant *S. aureus* (MRSA) activities of **85** (MIC = 0.025 μg/mL and 0.1 μg/mL, respectively) were 5–10-fold greater than those of vancomycin (MIC = 0.2 μg/mL and 0.625 μg/mL, respectively) [184]. Compounds **87** and **88** possessed antibacterial activity (MIC of 0.2 μg/mL for *S*. *aureus*, 0.625 μg/mL for MRSA, and 0.78 μg/mL for *B*. *subtilis*), similar to that of vancomycin. These isolated cyclic peptides (**85**–**88**) contain two unique piperazine moieties that have been found in over 140 compounds, with a broad spectrum of biological activities [184]. The reported mechanisms of action included membrane disruption, lipopolysaccharide transport binding, peptidoglycan lipid II precursor binding to affect cell wall synthesis, and an immunomodulation mechanism for the inhibition of pro-inflammatory cytokine release [185].

Nocathiacins I–III (**91**–**93**) are cyclic thiazolyl peptides identified in a culture of bacteria *Nocardia* sp. and have been found to be potent for Gram-positive pathogens (MICs ranging from 0.01 to 0.1 µg/mL), including representative isolates of penicillin-resistant *Streptococcus pneumoniae* (PRSP), vancomycin-resistant *Enterococcus faecium* (VREF), and MRSA. The MICs were minimally affected by human serum. In contrast, vancomycin MICs were 0.25–4.0 µg/mL for the same bacterial strains (64 µg/mL for the VRE strain) [186,187]. Excellent potency was observed against vancomycin-intermediate-resistant *S*. *aureus* strains, for which MICs were equivalent to those observed for susceptible strains (0.007 µg/mL). These compounds were also active against Gram-positive anaerobes such as *Clostridium difficile* and *Clostridium perfringens*, for which MICs compared favorably with MICs of the fluoroquinolone trovafloxacin [187]. Furthermore, the in vivo efficacy in a mouse model with systemic infection by *S*. *aureus* was obtained protective dose in 50% population (PD_50_) for **91**–**93** in a range of 0.62–0.89 mg/kg/day, respectively, compared to the reference antibiotic vancomycin (1.3 mg/kg/day) [186,188].

Rhodopeptin C1 (**97**), C2 (**98**), C3 (**99**), C4 (**100**), and B5 (**101**) were isolated from *Rhodococcus* species and belong to a family of antifungal cyclic lipotetrapeptides composed of a *β*-amino acid and three usual *α*-amino acids. Rhodopeptins (**97**–**101**) showed in vitro antifungal activity against *C*. *albicans* (MIC = 1.25–5 µg/mL) and *Cryptococcus neoformans* (MIC = 0.63–1.25 µg/mL) [189]. The results of SAR study revealed that a hydrophobic and bulky neutral amino acid (i.e., *γ*-methylleucine), and a basic amino acid moiety (lysine or ornithine) were indispensable structural motifs for antifungal activity. In addition, the structure of the lipophilic side chain did not have a crucial effect on the activity, as long as the total number of carbons ranged between 9 and 11 [190].

Rufomycins A (**102**) and B (**103**), also known as ilamycins [191], are highly interesting marine cyclopeptides [192], isolated from marine *Streptomyces* sp. [192,193,194,195,196,197,198]. Related peptides are characterized by their unusual amino acids and potent activity against a range of mycobacteria, including multidrug-resistant strains of *Mycobacterium tuberculosis*. Rufomycin A (**102**) and rufomycin B (**103**) have been reported to be highly active against Mycobacterium smegmatis, at 0.2 μg/mL and 0.5 μg/mL, respectively, and M. tuberculosis, at 0.1–0.4 μg/mL and 1–5 μg/mL, respectively, and also against strains resistant to other antibiotics (kanamycin, streptomycin, neomycin, and isonicotinic acid hydrazide). In addition, no significant toxicity was observed on four-week-old mice with intraperitoneal injection of **102** (LD_0_ 200 mg/kg and LD_100_ 360 mg/kg) [192]. In another study, rufomycin NBZ8 (**104**) was found to be the most active peptide, with an MIC of 0.02 μM (*M. tuber*. HR37v), similar to that of rifampicin [199]. The anti-tuberculosis activity results from the binding of the peptides to the *N*-terminal domain of the bacterial protease-associated unfoldase ClpC1, resulting in cell death [192,200].

Unnarmicins A (**114**) and C (**115**) are cyclic tetradepsipeptides produced by marine bacteria *Photobacterium* sp., comprising four amino acids and a 3-hydroxy FA. The main difference between them is in the length of the alkyl chain. These cyclodepsipeptides (**114** and **115**) were able to sensitize cells overexpressing azole drug pumps ScPdr5p, CaCdr1p, CgCdr1p, and CgPdh1p to sub-MIC concentrations of fluconazole without affecting the growth of CaCdr2p and CaMdr1p overexpressing cells. Both compounds (**114** and **115**) are potent inhibitors of rhodamine 6G efflux of CaCdr1p-expressing cells, with IC_50_ of 3.61 and 5.65 µM, respectively. Moreover, **114** and **115** inhibited in vitro CaCdr1p ATPase activity, with IC_50_ of 0.495 and 0.688 µM, respectively. They were also able to sensitize azole-resistant *C*. *albicans* clinical isolates to fluconazole. The ATPase activity of CaCdr1p, which drives active drug transport, was also inhibited to a similar extent by **114** and **115**, which could explain why 10-fold lower concentrations of both peptides were needed to inhibit the in vitro ATPase activity compared to the levels needed to inhibit the in vivo efflux of rhodamine 6G efflux using whole cells. These data suggest that the length of the alkyl side chain may significantly affect the different efficacies of these two compounds when using different biochemical assays, but from the in vitro ATPase assays it appears that both compounds have similar affinities to the target proteins and, therefore, also similar inhibitory activities [201].

A cyclic dodecadepsipeptide, valinomycin (**116**), isolated from marine bacteria *Streptomyces* sp., consists of polar groups oriented toward the central cavity, whereas the rest of the molecule is relatively nonpolar. It was shown to possess in vitro anti-parasitic activity against both *Trypanosoma brucei* (IC_50_ = 0.0032 μM) and *Leishmania major* (IC_50_ < 0.11 μM) [202].

**Table 2:** Antimicrobial cyclic peptides from marine bacteria.

Compound	Structure	Source	Antimicrobial Activity	Synthesis	References
Actinomycin V (**64**)	Cyclic pentapep-tide	*Streptomyces* sp.	MIC: MRSA (0.10–0.39 μg/mL), *S*. *epidermidis* (0.20–0.39 μg/mL), *E*. *faecium* (0.05–0.4 μg/mL), *E*. *faecalis* (0.20–0.39 μg/mL)	-	[203]
Bacillistatins 1 (**65**) and 2 (**66**)	Cyclic dodeca-despsipeptide	*Bacillus silvestris*	MIC: *S*. *pneumoniae* (1–2 μg/mL), PRSP (1 μg/mL), MDRSP (<0.5 μg/mL), *S*. *pyogenes* (1–8 μg/mL)	Total synthesis	[204,205]
Champacyclin (**67**)	Cyclic octapeptide	*Streptomyces champavatii*	40% inhibition of *E*. *amylovora* at 25 μM	-	[206]
Cyclomarins A-C (**68**–**70**)	Cyclic heptapepti-des	*Streptomyces* sp.	IC_50_: multidrug-resistant *Plasmodium falciparum* strains (0.25 μM), MIC: anti-tuberculosis activity (0.1 μM)	Total synthesis and analogues	[192,207]
Desotamide A (**71**) and desotamide B (**72**)	Cyclic hexapep-tide	*Streptomyces scopuliridis*	MIC: *S*. *pneumoniae* (13 µg/mL), *S*. *aureus* (16 µg/mL), MRSE (32 µg/mL)	Total synthesis	[208,209,210]
Fijimycins A–C (**73**–**75**) and etamycin A (**76**)	Cyclic octadepsi-peptides	*Streptomyces* sp.	MIC: three MRSA strains (4–32 µg/mL)	-	[211]
Halolitoralin A–C (**77**–**79**)	Cyclic tetrapepti-des	*Halobacillus litoralis*	MIC: *C*. *albicans* (20–30 µg/mL), and *T*. *rubrum* (25–40 µg/mL)	Total synthesis	[212,213]
Kocurin (**80**)	Cyclic thiazolyl heptadecapeptide	*Kocuria palustris*	MIC: MRSA (0.25 μg/mL)	-	[214]
Loloatins A-D (**81**–**84**)	Cyclic decapepti-des	Unknown bacteria from the Great Barrier Reef in Papua New Guinea	MIC: MRSA, VRE, PRSP (0.25–8 μg/mL)	Total synthesis	[215,216]
L-156,373 and three derivatives (**85**–**88**)	Cyclic heptapep-tides	*Streptomyces* sp.	MIC: *S*. *aureus*, MRSA, *B*. *subtilis* (0.025 to 1.25 μg/mL. Control vancomycin = 0.2 μg/mL, 0.625 μg/mL, 0.2 μg/mL, for each strain respectively), *Bacillus* Calmette-Guérin (1.25–12.5 μg/mL, Isoniazid (0.05 μg/mL) *C*. *albicans* (12.5 μg/mL), ketoconazole (0.016 μg/mL)	Total synthesis and analogues	[217,218]
Marthiapeptide A (**89**)	Tristhiazole-thiazoline cyclic peptide	*Marinactinospora thermotole-rans*	MIC: panel of Gram-positive bacteria (2.0–8.0 μg/mL)	Total synthesis	[219,220]
Mollemycin A (**90**)	Cyclic glycohexadepsipeptide-polyketide	*Streptomyces* sp.	IC_50_: *S*. *aureus* (10–50 nM), *S*. *epidermidis* (50 nM), and *B*. *subtilis* (10 nM), *E*. *coli* (10 nM), *P*. *aeruginosa* (50 nM), *M*. *bovis* (3.2 μM), antimalarial properties against drug sensitive strains (9 nM), MRPFC (7 nM)	-	[221]
Nocathiacins I (**91**), II (**92**), and III (**93**)	Cyclic thiazolyl peptides	*Nocardia* sp. or the fungi *Amicolaptosis* sp.	MIC: MRSA, MREF, FPRSP (0.01–0.1 μg/mL), vancomycin (0.25–4.0 μg/mL), in vivo efficacy of a systemic *S*. *aureus* infection mice model (PD_50_ = 0.62–0.89 mg/kg/day)	Semi-synthesis and analogues	[186,188,222,223,224,225,226]
Ohmyungsamycins A (**94**) and B (**95**)	Cyclic dodecapep-tides	*Streptomyces* sp.	MIC: Gram-positive and Gram-negative bacteria (8.50–34.0 μM)	Total synthesis	[227,228]
Pedein A (**96**)	Cyclic hexapeptide	*Chondromyces pediculatus*	MIC: *R*. *glutinis* (0.6 µg/mL), *S*. *cerevisiae*, *C*. *albicans* (1.6 µg/mL), and *U*. *maydis* (3.1 µg/mL)	-	[229]
Rhodopeptin C1 (**97**), C2 (**98**), C3 (**99**), C4 (**100**), and B5 (**101**)	Cyclic lipotetra-peptides	*Rhodococcus* sp.	MIC: *C*. *albicans* (1.25–5 µg/mL) and *C*. *neoformans* (0.63–1.25 µg/mL)	Total synthesis and analogues	[189,190,230]
Rufomycins A (**102**), B (**103**) and NBZ8 (**104**)	Cyclic heptapepti-des	*Streptomyces* sp.	MIC: *M. smegmatis* (0.2–5 μg/mL), *M. tuberculosis* (0.1–5 μg/mL), no toxicity by intraperitoneal injection **102**	Total synthesis and analogues	[192,193,194,195,196,197,198]
Salinamides A (**105**), B (**106**), and F (**107**)	Bicyclic polidepsi-peptides	*Streptomyces* sp.	MIC: *S*. *pneumoniae*, *S*. *pyogenes* (2–4 µg/mL, **105** and **106**) *S*. *aureus* (4 μM), MIC for compound **107***: E*. *faecalis* (12.5 μg/mL), *H*. *influenzae* (12.5 μg/mL), *N*. *gonorrhoeae* (25 μg/mL), *E*. *cloacae* (50 μg/mL), and *E*. *coli* (0.20 μg/mL)	Total synthesis	[231,232,233]
Streptocidins C (**108**) and D (**109**)	Cyclic homodeca-peptide	*Streptomyces* sp.	MIC: *B*. *subtilis* (3 µg/mL), *S*. *aureus* (3–10 µg/mL), *S*. *viridochromogenes* (1–3 µg/mL), and *Streptomyces* (3–10 µg/mL)	Total synthesis	[234,235]
Theopalauamide A (**110**)	Bicyclic glycodode-capeptide	Eubacteria symbiosis sponge *Theonella swinhoei*	*C*. *albicans* (10 µg/disk)	-	[236]
Thiocoraline (**111**)	Bicyclic octadepsipeptide	Actinomycete	MIC: *S*. *aureus* (0.05 µg/mL), *B*. *subtills* (0.05 µg/mL), *M*. *luteus* (0.03 µg/mL).	Total synthesis and analogues	[237,238,239]
TP-1161 (**112**)	Cyclic thiopeptide	*Nocardiopsis* sp.	MIC: *S*. *aureus* (0.5–32 μg/mL), *S*. *haemolyticus* (0.5–1 μg/mL), *S*. *epidermidis* (0.5–4 μg/mL), *E*. *faecalis* (1 μg/mL), *E*. *faecium* (0.5 μg/mL), VREF (1 μg/mL), *S*. *pneumoniae* (0.5 μg/mL), *S*. *agalactiae* (0.5 μg/mL)	-	[240]
Tumescenamide C (**113**)	Cyclic lipopenta- depsipeptide	*Streptomyces* sp.	*S*. *coelicolor*, *S*. *lividans* (inhibition zone 3.0 mg/paper disk)	Total synthesis and analogues	[241,242]
Unnarmicin A (**114**) and C (**115**)	Cyclic tetradepsi-peptides	*Photobacte-rium* sp.	IC_50_: Fluconazole-resistant *C. albicans* isolates (0.495–0.688 μM)	Total synthesis of analogue	[201]
Valinomycin (**116**)	Cyclic dodecadep-sipeptide	*Streptomyces* sp.	IC_50_: *T*. *brucei* (0.0032 μM) and *L*. *major* (<0.11 μM)	Total synthesis and analogues	[202,243,244,245]

IC_50_ (half maximal inhibitory concentration); FPRSP (fully penicillin-resistant *Streptococcus pneumoniae*); MDRSP (multidrug-resistant *Streptococcus pneumoniae*); MIC (minimum inhibitory concentration); MREF (multidrug-resistant *Enterococcus faecium*); MRPFC (multidrug-resistant *Plasmodium falciparum* clones); MRSA (methicillin-resistant *Staphylococcus aureus*); MRSE (methicillin-resistant *Staphylococcus epidermidis*); PD_50_ (protective dose in 50% population); PRSP (penicillin-resistant *Streptococcus pneumoniae*); VRE (vancomycin-resistant *Enterococci*), VREF (vancomycin-resistant *Enterococci faecalis*). *Bacillus subtilis (B. subtilis*); *Candida albicans* (*C. albicans)*, *Cryptococcus neoformans* (*C. neoformans*); *Enterobacter cloacae* (*E*. *cloacae*); *Enterococcus* species (*E*. *faecalis*, *E*. *faecium*); *Erwinia amylovora* (*E*. *amylovora*); *Escherichia coli* (*E. coli*); *Haemophilus influenzae* (*H*. *influenzae*); *Leishmania major* (*L*. *major*); *Micrococcus luteus* (*M*. *luteus*); *Mycobacterium bovis* (*M*. *bovis*); *Neisseria gonorrhoeae* (*N*. *gonorrhoeae*); *Pseudomonas aeruginosa* (*P*. *aeruginosa*); *Rhodotorula glutinis* (*R*. *glutinis*); *Saccharomyces cerevisiae* (*S*. *cerevisiae*); *Staphylococcus* species (*S*. *aureus*, *S*. *epidermidis*, *S*. *haemolyticus*); *Streptococcus* species (*S*. *agalactiae*, *S*. *pneumoniae*, *S*. *pyogenes*); *Streptomyces* species (*S*. *coelicolor*, *S*. *lividans*, *S*. *viridochromogenes*); *Trichophyton rubrum (T. rubrum); Trypanosoma brucei* (*T*. *brucei*); *Ustilago maydis* (*U*. *maydis*).

### 2.3. Cyanobacteria-Produced Cyclic Peptides

**Figure 5:**
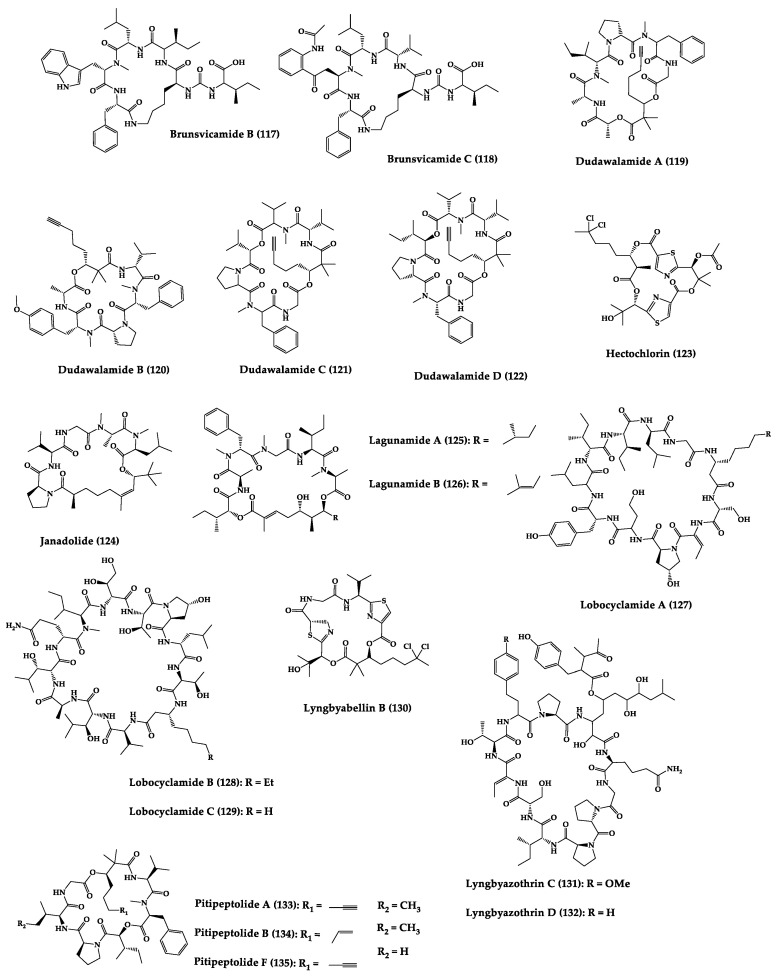

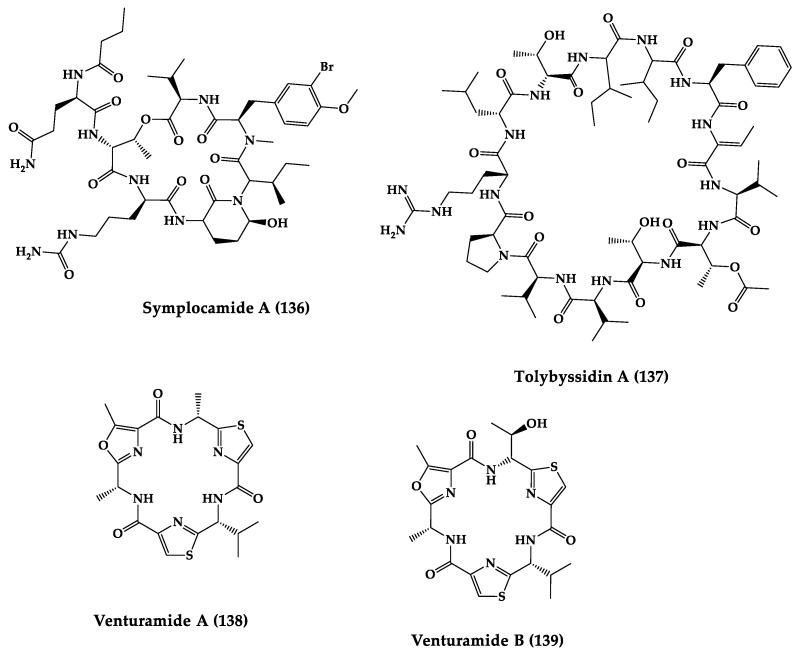
Chemical structures of cyclic peptides from cyanobacteria (**117**–**139**).

Cyanobacteria are extensively distributed around the world, as some of the primogenital oxygenic and photosynthetic aquatic prokaryotes. It has been found that structurally diverse natural marine compounds with broad biological activities can be obtained from marine cyanobacteria [246]. Secondary metabolites of marine cyanobacteria are produced as chemical defenses to improve the adaptability of marine cyanobacteria to various marine environments characterized by hypersalinity, high pressure, and complexity [247]. In the last decade, there has been an outstanding increase in the discovery of peptides derived from marine cyanobacteria with peculiar chemical structures [248]. In this section, 23 cyclic peptides isolated from cyanobacteria (**117**–**139**) are reported (Figure 5 and Table 3). Among these, nine cyclic peptides have been described with antibacterial activity, as well as six with antifungal and eight with antiparasitic activity. 

Dudawalamides A–D (**119**–**122**) were isolated from a Papua New Guinean field collection of the cyanobacterium *Moorea producens*, consisting of the amino acids glycine, *N*-methylphenylalanine, *N*-methylisoleucine, proline, alanine, and lactic acid. Dudawalamides **119** and **122** showed the most potent activities against *P. falciparum*, with IC_50_ values of 3.6 and 3.5 μM, respectively. However, weaker activities were observed against both *Trypanasoma cruzi* and *Leishmania donovani*, whereas **122** was relatively potent against *L. donovani* (IC_50_ = 2.6 μM). Dudawalamides B (**120**) and C (**121**) were significantly less potent than **119** and **122** against *P. falciparum*, and **120** also showed decreased potency to the other two parasites [249].

The most relevant antimicrobial cyclic peptides have been highlighted, including janadolide (**124**), isolated from an *Okeania* sp. marine cyanobacteria, which is a cyclic polyketide–peptide hybrid possessing a *tert*-butyl group, and showed in vitro antitrypanosomal activity with an IC_50_ of 47 nM without cytotoxicity against human cells at 10 μM [250].

Lagunamides A (**125**) and B (**126**) were isolated from the marine cyanobacteria *Lyngbya majuscula* obtained from Pulau Hantu Besar, Singapore. Both are cyclic pentadepsipeptides, and display in vitro antimalarial properties, with IC_50_ of 0.19 µM and 0.91 µM, respectively, when tested against *Plasmodium falciparum*. Moreover, **125** and **126** displayed antiswarming activity when tested at 100 ppm against the Gram-negative bacterial strain *Pseudomonas aeruginosa*, which exerted 62% for **125** and 56% for **126** compared to control. Interestingly, the only structural difference between **125** and **126** is an additional olefinic group between C_40_–C_41_ in **126**, and this minor difference is reflected in the enhanced antimalarial activity observed in **125** [251].

Pitipeptolides A (**133**) and B (**134**), as well as one homologue of **133**, pitipeptolide F (**135**), are cyclodepsipeptides isolated from a population of the marine cyanobacterium *Lyngbya majuscula* sponge symbiosis collected at Piti Bomb Holes, Guam. Pitipeptolide F (**135**) showed the highest potency in the disc diffusion assay against *M. tuberculosis*. The findings lead to the following SAR conclusions: (1) *N*-methylation in the phenylalanine unit is important for both cytotoxic and antibacterial activities; (2) the *π* system in the FA unit is one of the essential features for cytotoxic activity in mammalian cells, but it is not essential for antibacterial activity; (3) decreasing the hydrophobicity of certain units, an increased antibacterial potency was observed for some compounds [252,253].

**Table 3:** Antimicrobial cyclic peptides from marine cyanobacteria.

Compound	Structure	Source	Antimicrobial Activity	Synthesis	References
Brunsvica-mide B (**117**) and C (**118**)	Cyclic hexapep-tides	Sponges symbiosis *Tychonema* sp.	IC_50_: *M. tuberculosis* protein tyrosine phosphatase B (7.3–8.0 µM)	Total synthesis of analogues	[254,255,256,257]
Dudawala-mides A-D (**119**–**122**)	Cyclic depsipep-tides	*Lyngbya* sp.	IC_50_: *P*. *falciparum* (2.7–7.7 μM), *L*. *donovani* (2.6–25.9 μM), and **116** against *T*. *cruzi* (7.3 μM)	-	[249]
Hectochlorin (**123**)	Cyclic depsipep-tide	*Lyngbya majuscula*	*C*. *albicans* (10 µg/disk: 11 mm)	Total synthesis	[258,259]
Janadolide (**124**)	Cyclic polyketi-depeptide hybrid	*Okeania* sp.	IC_50_: Antitrypanosomal activity (47 nM)	Total synthesis	[250,260]
Lagunamides A (**125**) and B (**126**)	Cyclic penta-depsipep-tides	*Lyngbya majuscula*	IC_50_: *P*. *falciparum* (0.19–0.91 µM), *P*. *aeruginosa* (antiswarming activity at 100 ppm, exerted 62% for **119** and 56% for **120**)	Total synthesis and analogues	[251,261,262,263,264,265]
Lobocycla-mides A-C (**127**–**129**)	Cyclic dodeca-peptide	Sponges symbiosis *Lyngbya confervoides*	Antifungal activity: FRFCA (150 µg/disk: **121** = 7 mm inhibition zone diameters; **122** = 8 mm; **121** = 10 mm) and *C*. *glabrata* (150 µg/disk: **122** = 6 mm; **123** = 8 mm)	-	[266,267]
Lyngbya- bellin B (**130**)	Cyclic hexa- depsipeptide	Sponges symbiosis *Lyngbya majuscula*	*C*. *albicans* (100 µg/disk: 10.5 mm)	Total synthesis and analogues	[268,269,270,271]
Lyngbyazo-thrins C (**131**) and D (**132**)	Cyclic undeca-peptides	Sponges symbiosis *Lyngbya* sp.	*B*. *subtilis* (25 µg/disk: 18 mm), *E*. *coli* (100 µg/disk: 15 mm), *P*. *aeruginosa* (100 µg/disk: 8 mm), *S*. *marcescens* (200 µg/disk: 8 mm)	-	[272]
Pitipeptolides A (**133**), B (**134**) and F (**135**)	Cyclic hexa-depsipeptides	Sponges symbiosis *Lyngbya majuscula*	*M*. *tuberculosis* (10 µg/disk: 9–14 mm), streptomycin (10 µg/disk: 40 mm)	Semi-synthesis	[252,253,273,274]
Symplocamide A (**136**)	Cyclic lipodepsi-peptide	*Symploca* sp.	IC_50_: *P. falciparum* (0.95 µM), *T. cruzi* (>9.5 µM), *L. donovani* (>9.5 µM)	Total synthesis	[275,276]
Tolybyssidin A (**137**)	Cyclic trideca-peptides	*Tolypothrix byssoidea*	MIC: *C*. *albicans* (32 µg/mL), miconazole (8 µg/mL)	-	[277]
Venturamides A (**138**) and B (**139**)	Cyclic hexa-peptides	Sponges symbiosis *Oscillatoria* sp.	IC_50_: *P. falciparum* (5.6–8.2 µM), *T. cruzi* (14.6–15.8 µM), *L. donovani* (>19–20 µM)	Total synthesis	[278,279]

ED_100_ (effective dose in 100% of population); FRFCA (fluconazole-resistant fungi *Candida albicans*); IC_50_ (half maximal inhibitory concentration); MIC (minimum inhibitory concentration), MIC_50_ (concentration at which 50% of the strains were inhibited). *Bacillus subtilis* (*B*. *subtilis*); *Candida* species (*C*. *albicans*, *C*. *glabrata*, *C*. *tropicalis*); *Colletotrichum gloeosporioides* (*C*. *gloeosporioides*); *Escherichia coli* (*E*. *coli*); *Fusarium oxysporum* (*F*. *oxysporum*); *Leishmania* *donovani* (*L*. *donovani*); *Mycobacterium tuberculosis (M. tuberculosis); Plasmodium falciparum* (*P*. *falciparum*); *Pseudomonas aeruginosa* (*P*. *aeruginosa*); *Rhizoctonia* *solani* (*R*. *solani*); *Rhodotorula rubra* (*R*. *rubra*); *Saccharomyces cerevisiae* (*S*. *cerevisiae*); *Sclerotium rolfsii* (*S*. *rolfsii*); *Serratia marcescens* (*S*. *marcescens*); *Trypanosoma cruzi*, (*T*. *cruzi*).

### 2.4. Fungi-Produced Cyclic Peptides

**Figure 6:**
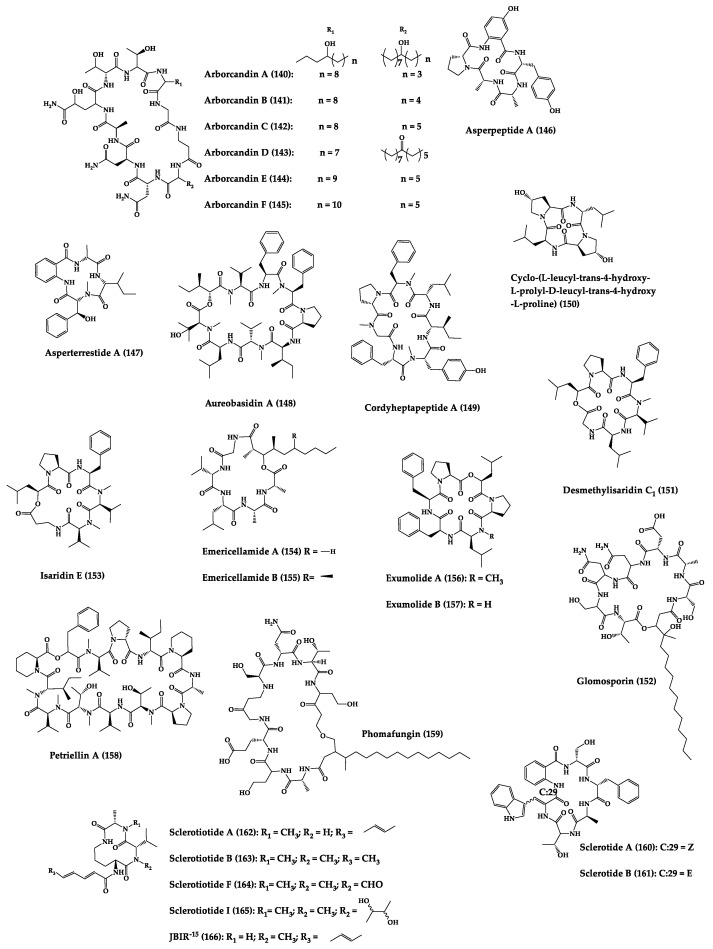
Chemical structures of cyclic peptides from fungi (**140**–**166**).

Fungal marine microorganisms are a valuable source of bioactive natural products. Hundreds of secondary metabolites obtained from marine fungal strains have revealed potent pharmacological and biological activities [280]. As an example, in 1948, one of the most revolutionary antibiotics, cephalosporin, used to treat typhoid fever, was isolated for the first time from cultures of a fungus, *Cephalosporium acremonium*, from a sewer located on the Italian island of Sardinia [281]. In this section, 27 cyclic peptides isolated from fungi (**140**–**166)** are described (Figure 6 and Table 4). Among these, eight cyclic peptides have been described with antibacterial activities, as well as nine with antifungal, eight with parasitic, and none with antiviral activities. 

Arborcandins A–F (**140**–**145**), isolated from the culture broth of a filamentous of unknown fungi, demonstrated 1,3-*β*-glucan synthase inhibitory activity [282]. They are cyclic lipopentapeptides, which are structurally different from known glucan synthase inhibitors such as echinocandins, isolated from soil fungi. The 1,3-*β*-glucan synthases of *C*. *albicans* and *Aspergillus fumigatus* were inhibited by **140**–**145**, with IC_50_ ranging from 0.012 to 3 µg/mL. The apparent competitive inhibition constant values of arborcandin C (**142**) for *C*. *albicans* and *A*. *fumigatus* were 0.12 µM and 0.016 µM, respectively. The inhibition against these two 1,3-*β*-glucan synthases by **140** was noncompetitive. Compounds **140**–**145** exhibited fungicidal activity against *Candida* spp. with MIC ranging from 0.25 to 8 µg/mL. The growth of *A*. *fumigatus* was suppressed by **140**–**145**, with concentrations ranging from 0.063 to 4 µg/mL. Among them, arborcandin C (**142**), D (**143**), and F (**145**) exhibited stronger glucan synthase inhibitory activity. It seems that arborcandins comprising longer alkyl side chains had stronger activity. Arborcandin D (**143**), in which the hydroxyl residue in the alkyl side chain is replaced with a ketone, showed a much weaker activity. This suggests that the hydroxyl residue may have an important role in the activity of these peptides [283].

Aureobasidin A (**148**) is a cyclic octadepsipeptide produced by *Aureobasidium pullulans* and showed in vitro antifungal activity, especially against *C*. *albicans* (MIC = 0.05 µg/mL) and *C*. *neoformans* (MIC = 0.78 µg/mL), which were more than ten times lower than the MIC of amphotericin B [284]. No signs of toxicity were observed for **148** when administered intraperitoneally once to mice at a dose of 200 mg/kg [285].

A cyclic lipooctadepsipeptide isolated from a *Phoma* sp. phomafungin (**159**), containing a 28-member ring with eight amino acids and a *β*-hydroxy-*ɤ*-methyl-hexadecanoic acid, displayed a broad spectrum of antifungal activity against *Candida* spp., *Aspergillus fumigatus* and *Trichophyton mentagrophytes*, with MIC of 2–8 µg/mL, and toxicity to mice was found at 25 mg/kg. Moreover, **159** had no activity against *C*. *tropicalis* [286].

**Table 4:** Antimicrobial cyclic peptides from marine fungi.

Compound	Structure	Source	Antimicrobial Activity	Synthesis	References
Arborcandins A–F (**140**–**145**)	Cyclic lipopentapep-tides	Unknown filamentous fungi	MIC: *Candida* spp. (0.25–8 µg/mL), *A*. *fumigatus* (0.063–4 µg/mL)	-	[283]
Asperpeptide A (**146**)	Cyclic pentapeptide	*Aspergillus* sp.	MIC: *B*. *cereus*, *S*. *epidermidis* (12.5 μM)	-	[287]
Asperterrestide A (**147**)	Cyclic tetrapeptide	*Aspergillus terreus*	IC_50_: H_1_N_1_, H_3_N_2_ influenza strains (8.1–15 μM), ribavirin (0.41–20.2 μM)	Total synthesis	[288,289]
Aureobasidin A (**148**)	Cyclic octadepsipep-tide	*Aureobasidium pullulans*	MIC: *C*. *albicans* (0.05 µg/mL) and *C*. *neoformans* (0.78 µg/mL)	Total synthesis and analogues	[284,285,290,291]
Cordyhep- tapeptide A (**149**)	Cyclic heptapeptide	*Cordyceps* sp.	IC_50_: Antimalarial activity (3.8 μM)	Total synthesis	[292,293,294]
Cyclo-(L-leucyl-*trans*-4-hydroxy-L-prolyl-D-leucyl-*trans*-4- hydroxy-L-proline) (**150**)	Cyclic tetrapeptide	*Phomopsis* sp. and *Alternaria* sp.	MIC: *G*. *graminis* (220 µg/mL), *R*. *cerealis* (160 µg/mL), *H*. *sativum* (130 µg/mL), *F*. *graminearum* (250 µg/mL)	-	[295]
Desmethyl- isaridin C1 (**151**) and isaridin E (**152**)	Cyclic hexadepsipeptides	Bryozoan-derived fungus *Beauveria felina*	*E*. *coli* (MIC = 8–16 µg/mL)	-	[296]
Emericellamides A (**153**) and B (**154**)	Cyclic pentadepsi-peptide	*Emericella* sp.	MIC: MRSA (3.8 and 6.0 µM, respectively)	Total synthesis	[297,298,299,300]
Exumolides A (**155**) and B (**156**)	Cyclic hexadepsipeptides	*Scytalidium* sp.	Antimicroalgal activity against chlorophyte *Dunaliella* (reduction in growth of 27–33% at 20 µg/mL)	Total synthesis	[301,302]
Glomosporin (**157**)	Cyclic lipohepta-depsipeptide	*Glomospora* sp.	MIC: *A*. *fumigatus* (16 µg/mL)	-	[303]
Petriellin A (**158**)	Cyclic dodecadepsi-peptide	*Petriella sordida*	MIC: *A*. *furfuraceus* (5 µg/mL), *S*. *fimicola* (52 µg/mL)	Total synthesis	[304,305]
Phomafungin (**159**)	Cyclic lipoocta-depsipeptide	*Phoma* sp.	MIC: *Candida* spp., *A*. *fumigatus*, *T*. *mentagrophytes* (2–8 µg/mL)	-	[286]
Sclerotides A (**160**) and B (**161**)	Cyclic hexapeptides	*Aspergillus sclerotiorum*	MIC: *C*. *albicans* (7.0 µM and 3.5 µM, respectively), *P*. *aeruginosa* (35.3 µM for **156**)	Total synthesis	[306,307]
Sclerotiotides A (**162**), B (**163**), F (**164**), I (**165**) and JBIR-15 (**166**)	Cyclic tripeptides	*Aspergillus sclerotiorum*	MIC: *C*. *albicans* (3.8–30 µM)	-	[308]

IC_50_ (half maximal inhibitory concentration); MIC (minimum inhibitory concentration); MRSA (methicillin-resistant *Staphylococcus aureus*). *Ascobolus furfuraceus* (*A*. *furfuraceus*); *Aspergillus fumigatus* (*A*. *fumigatus*); *Bacillus cereus* (*B. cereus*), *Candida albicans* (*C*. *albicans*); *Cryptococcus neoformans* (*C*. *neoformans*); *Escherichia coli* (*E*. *coli*); *Gaeumannomyces graminis* (*G*. *graminis*); *Fusarium graminearum* (*F. graminearum*); *Helminthosporium sativum* (*H*. *sativum*); *Pseudomonas aeruginosa* (*P*. *aeruginosa*); *Rhizoctonia cerealis* (*R*. *cerealis*); *Sordaria fimicola* (*S*. *fimicola*); *Staphylococcus epidermidis* (*S. epidermidis); Trichophyton* *mentagrophytes* (*T*. *mentagrophytes*).

### 2.5. Other Marine Invertebrate-Produced Cyclic Peptides

**Figure 7:**
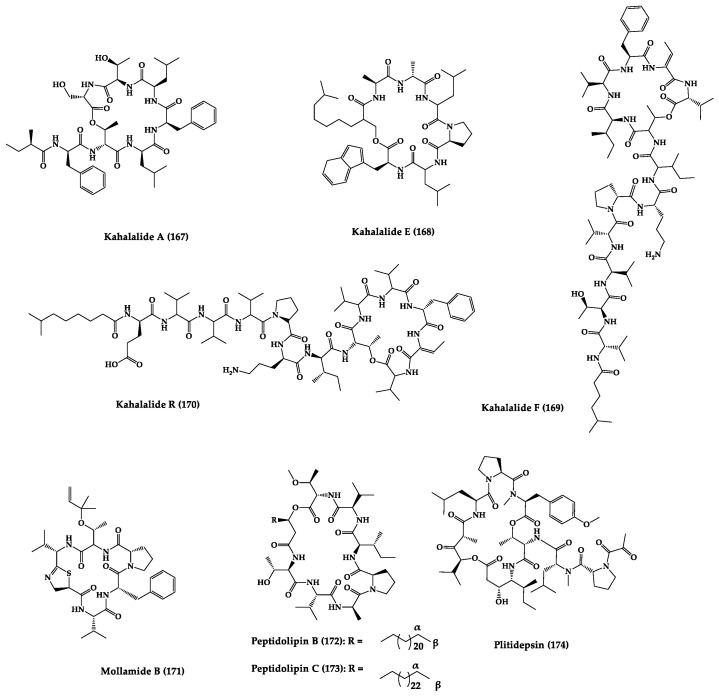
Chemical structures of cyclic peptides from other marine invertebrates (**167**–**174**).

Other marine invertebrates such as ascidians and sea hare are sources of cyclic peptides. Ascidians belong to the class of the subphylum Tunicata, with more than 3000 described species [309]. They can be found in diverse ecological niches, from deep-sea waters to shallow waters. Moreover, the development of culture-independent methods has provided thorough evidence on the microbial variety of sea squirts [310]. Sea hares are considered to be shell-less mollusks comprising soft bodies with a soft inner shell made of protein [311]. Sea hares do not have a well-developed shell to give them mechanical protection from predation and are slow, so they cannot use armor or speed to avoid fast predators such as crabs or fish. They have developed other means of protection, including encryption, large size, and an impressive array of chemical defenses [311,312]. However, the most effective defense mechanism displayed by these organisms is the chemical and behavioral one, releasing a purple ink and opaline when attacked by predators [313,314]. In this section, eight cyclic peptides (**167**–**174**) isolated from other marine invertebrates are reported (Figure 7 and Table 5). Among them, six cyclic peptides have been described as having antibacterial activities, four with antifungal, five with parasitic, and two with antiviral activities.

Kahalalides (**167**–**170**), isolated from green alga metabolites that are eaten by the sacoglossan mollusk, *Elysia rufescens*. They are a family of cyclic depsipeptides with variable size and peptide series, ranging from C_31_ to C_77_ and carrying different FA chains [315]. Kahalalide A (**167**) has been demonstrated in vitro to inhibit 83% of the growth of *M*. *tuberculosis* at 12.5 μg/mL [316]. Although kahalalides F (**169**) and R (**170**) were inactive toward Gram-positive and Gram-negative bacteria, **163** exhibited antifungal activity with IC_50_ of 3.02 μM against *C*. *albicans*, 1.53 μM against *C*. *neoformans*, and 3.21 μM against *A*. *fumigatus* [317]. In an agar diffusion assay, **169** also exhibited antifungal activity at a level of 5 μg/disk against the plant pathogens *Cladosporium herbarum* and *Cladosporium cucumerinum*, with inhibition zones of 17 and 24 mm, respectively (with the positive control nystatin of 19 and 39 mm, respectively) [318]. Furthermore, in an agar diffusion assay, kahalalide R (**170**) at 5 µg/disk, showed antifungal activity against the plant pathogens *Cladosporium herbarum* and *Cladosporium cucumerinum*, with inhibition zone of 16 and 24 mm, respectively (nystatin at 19 and 39 mm, respectively). Kahalalide E (**168**) exhibited activity against HSV II at 5 µg/mL [316]. Kahalalide F (**169**) also exhibited in vitro antiviral activity at 0.5 μg/mL (95% reduction) with HSV II using mink lung cells, and it exhibited selective activity against some of the Acquired Immunodeficiency Syndrome (AIDS) opportunistic infections [316,319]. The in vitro activity of **169** against promastigote and amastigote stages of *Leishmania* was also tested, affording values of 6.13 μM against *L. donovani* (promastigote), 8.31 μM against *L. pifanoi* (promastigote), 29.53 μM against *L. pifanoi* (amastigotes) [320]. Bioassays showed that compound **170** exerted equal or greater cytotoxic activity than **169** [318]. Regarding SAR studies, it was found that the free serine and threonine side chains, as well as the constrained depsipeptide framework, were important features for biological activity against *M*. *tuberculosis*. In addition, it was emphasized that the methylbutyrate side chain is flexible and can be replaced by other hydrophobic groups, as evidenced by increased activity with hexanoate [318]. Kahalalide F (**169**) was not sensitive to side chain substitutions in almost every residue, and it was possible to find a distinct side chain that could preserve or even improve the activity. A more hindered replacement in each side chain was able to improve the activity by enhancing the hydrophobicity at any point on the molecule, with the solubility in water being a limiting factor [318].

Recently, Kris M. Whit et al. [321] proved that a cyclic depsipeptide, plitidepsin (**174**), isolated from tunicate *Aplidium albicans*, inhibited SARS-CoV-2 in an antiviral screening assay in Vero E6 cells with 90% maximum inhibitory concentration (IC_90_) of 1.76 nM and an IC_90_ value of 0.88 nM in human cells, with limited toxicity in cell culture. These results demonstrated that **174** is more potent than remdesivir tested in the same cell line by a factor of 27.5. Using a drug-resistant mutant, the antiviral activity of **174** against SARS-CoV-2 was shown to be mediated through inhibition of the known target eukaryotic translation elongation factor 1A. The in vivo efficacy of **174** for treatment in two mouse models of SARS-CoV-2 infection was also demonstrated, with a reduction of viral replication in the lungs by two orders of magnitude using prophylactic treatment [321].

**Table 5:** Antimicrobial cyclic peptides from other marine invertebrates.

Compound	Structure	Source	Antimicrobial Activity	Synthesis	References
Kahalalides A (**167**), E (**168**), F (**169**), and R1 (**170**)	Cyclic depsipep-tides	Green alga metabolites Sacoglossan mollusk *Elysia rufescens*	*M*. *tuberculosis* (inhibited 83% at 12.5 μg/mL), *C*. *albicans* (IC_50_ = 3.02 μM), *C*. *neoformans* (IC_50_ = 1.53 μM), *A*. *fumigatus* (IC_50_ = 3.21 μM), *C*. *herbarum* and *C*. *cucumerinum* at 5 μg/disk with inhibition zones of 17 and 24 mm, respectively), *L*. *donovani* promastigote (IC_50_ = 13 μM), *L*. *pifanoi* promastigote (IC_50_ = 13 μM), *L*. *pifanoi* amastigotes (IC_50_ = 29.53 μM)	Total synthesis and analogues	[316,317,318,319,320,322,323,324,325]
Mollamide B (**171**)	Cyclic hexapep-tide	Tunicate *Didemnum mole*	IC_50_: *P*. *falciparum* clones (2.0–2.1 µg/mL), IC_90_: *L*. *donovani* (18 and 35 µg/mL, respectively), EC_50_: HIV-1 in human peripheral blood mononuclear cells (48.7 µM)	Total synthesis of analogues	[326]
Peptidolipins B (**172**) and C (**173**)	Cyclic lipo- heptapeptide	Marine *Nocardia* sp. cultivated from ascidian *Trididemnum orbiculatum*	MSSA, MRSA (MIC > 64 μg/mL)	-	[327]
Plitidepsin (**174**)	Cyclic depsipep-tide	tunicate *Aplidium albicans*	SARS-CoV-2 in human cell line (IC_50_ = 0.73, CC_50_ = 200 nM) and in pneumocyte-like cells (IC_50_ = 1.62, CC_50_ = 65.43 nM)	Total synthesis and analogues	[321,328,329]

CC_50_ (50% cytotoxic concentration); HIV (human immunodeficiency virus); EC_50_ (effective concentration in 50% of population); IC_50_ (half maximal inhibitory concentration); IC_90_ (maximum inhibitory concentration in 90% population); MIC (minimum inhibitory concentration); MRSA (methicillin-resistant *Staphylococcus aureus*); MSSA (methicillin-susceptible *Staphylococcus aureus*); SARS-CoV-2 (severe acute respiratory syndrome coronavirus 2). *Aspergillus fumigatus* (*A*. *fumigatus*); *Candida albicans* (*C*. *albicans*); *Cladosporium* species (*C*. *cucumerinum*, *C. herbarum*); *Cryptococcus neoformans* (*C*. *neoformans*); *Leishmania* species (*L*. *donovani*, *L*. *pifanoi*); *Mycobacterium tuberculosis* (*M*. *tuberculosis*); *Plasmodium falciparum* (*P*. *falciparum*).

## 3. Synthetic Methods to Obtain Cyclic Peptides

Cyclic peptides are oligopeptide chains which undergo intramolecular cyclization linking one end of the peptide and the other end with an amide bond, or other chemically stable bonds, such as lactone, ether, thioether, disulfide, and so on [330]. The choice of cyclization site and the order of residue coupling are fundamental aspects to be analyzed in the planning of a possible synthetic pathway [331].

**Figure 8:**
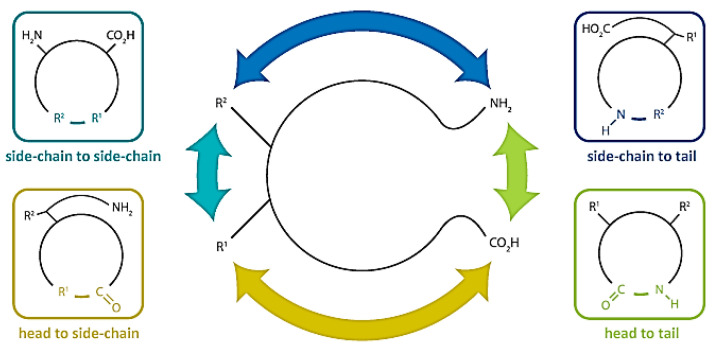
Schematic representation of the cycling possibilities.

Depending on the functional groups present, peptides can be cyclized in different ways, as shown in Figure 8. Conventional approaches typically used to synthesize mono- and polycyclic peptides consist of head to tail, side-chain to tail, head to side-chain, and side-chain to side-chain, as well disulfide formation [332]. The challenge of cyclization of peptides led to the search for different synthetic methodologies with the incorporation of different organic structures to generate macrocycles [333]. Regarding the synthesis of cyclic peptides, ring closure can occur in the ester bond (macrolactonisation), rather than the formation of the amide bond (macrolactamisation) [105].

Trimerization, cyclodimerization [334], epimerization at the cyclization site with non-glycine/proline C-terminus [335], and formation of oligomers, resulting from intermolecular reactions, are major concerns regarding peptide synthesis. In linear precursors, the *N* and *C* termini are far from each other due to a more stable all-*trans* configuration of the amide bonds and, as a result, are less likely to react intramolecularly to cyclize [331]. Esterification is another synthetic challenge for a molecule with more than one ester group, with it being difficult to find selectivity that facilitates saponification when necessary [336].

A crucial step is the formation of the peptide bond, which typically requires the activation of the carboxylic acid using a peptide coupling agent [337]. It should be noted that, due to the requirement for full retention of chiral amino acids integrity, mild conditions are needed and can be challenging in the coupling reactions [338]. In terms of acid carboxilic activator, cyanuric chloride has been used for the preparation of acyl chlorides, amides and peptides [339]. A cyanuric chloride derivative, 2-chloro-4,6-dimethoxy-1,3,5-triazine (CDMT), is a coupling agent that reacts with carboxylic acids to form reactive esters and it can strongly acylate amines and less nucleophilic alcohols [340]. The activation of the carboxylic acid is performed in the presence of a base, such as *N*-methylmorpholine (NMM). In situ NMM and CDMT form the intermediate 4-(4,6-dimethoxy-1,3,5-triazin-2-yl)-4-methyl-morpholinium chloride (DMTMM), which can be used independently as a coupling reagent [341]. If used alone, DMTMM does not need carboxylic acid pre-activation. The coupling efficiency of DMTMM in solid-phase peptide synthesis (SPPS) is comparable to that of benzotriazole-1-yloxytripyrrolidinephosphonium hexafluorophosphate (PyBOP), with a low level of racemization [342].

*N*,*N*-4-Dicyclohexylcarbodiimide (DCC) is extensively used in *tert*-butyloxycarbonyl (Boc)/benzyl (Bzl)-protecting group peptide synthesis, because the 1,3-dicyclohexyl urea (DCU) by-product is easily removed from the reaction vessel in the presence of trifluoroacetic acid (TFA) during the Boc-deprotection protocol. In the 9-fluorenylmethyloxycarbonyl (Fmoc)/*tert*-butyl (*t*-But) chemistry, diisopropylcarbodiimide (DIC) gives rise to a more *N*,*N*-dimethylformamide (DMF)-soluble urea by-product, and is therefore highly recommended [343]. *N*-(3-Dimethylaminopropyl)-*N*′-ethylcarbodiimide hydrochloride (EDC) is widely used in the solution phase, as it generates a urea by-product which can be easily removed from the reaction medium by extraction with water [344]. Another important water-soluble carbodiimide is *N*-cyclohexyl-*N*′-(2-morpholinoethyl)carbodiimide-methyl-*p*-toluenesulfonate (CMC) [345,346]. A major drawback of the cabodiimide procedure is the dehydration of side-chain carboxamides of asparagine—and glycine—residues to the corresponding nitriles. This problem is completely avoided when using carbodiimides in combination with additives like hydroxylamine derivatives, such as 1-hydroxybenzotriazole (HOBt) or 7-aza-1-hydroxybenzotriazole (HOAt) [347,348,349]. (1-[Bis(dimethylamino)methylene]-1*H*-1,2,3-triazolo [4,5-*β*]pyridinium 3-oxide hexafluorophosphate (HATU) is a reagent used in peptide coupling chemistry to generate an active ester from a carboxylic acid. HATU is used along with Hünig’s base, *N*,*N*-diisopropylethylamine (DIPEA), or triethylamine (TEA) to form amide bonds. Typically, DMF is used as solvent, although other polar aprotic solvents can also be used [350]. HATU is similar to 2-(1*H*-benzotriazol-1-yl)-1,1,3,3-tetramethyluronium hexafluorophosphate (HBTU), but reacts faster with less epimerization during coupling. HATU is preferred to HBTU in most rapid coupling protocols [351]. 

1-Cyano-2-ethoxy-2-oxoethylidenaminooxy)dimethylamino-morpholino-carbenium hexafluorophosphate (COMU) is a highly efficient coupling reagent that produces yields as good as or even better than that of HATU [352]. The dimethylmorpholino skeleton in COMU affords high solubility in DMF [353], affording solutions that are more concentrated than with HBTU or HATU solutions. In contrast to HATU and HBTU, COMU only requires one equivalent of base in coupling reactions. It produces very low racemization and is a preferred reagent for fragment coupling [354]. To prevent polymerization of the amino acid once it is activated, the protection of the amine and carboxylic acid functional groups are mandatory issues. The most common amino-protecting groups for SPPS are the Fmoc and the Boc groups used in the Fmoc/*tert*-but and Boc/Bzl strategies, respectively [355].

### Total Synthesis of Natural Cyclic Peptides

In recent decades, several antimicrobial cyclic peptides have been approved for clinical use [109]. In fact, some of the antibiotics available on the market are cyclic antimicrobial peptides like caspofungin, vancomycin, daptomycin, cyclosporine, polymyxin B, colistin, tyrocidin, gramicidin, batracin, and daptomycin [356]. Antimicrobial peptides are attracting renewed interest as potential candidates for therapeutic antibiotics [357]. However, the amounts obtained from natural sources and from synthetic procedures have presented difficulties in terms of cyclic peptides reaching the drug discovery pipeline, in turn presenting a major challenge for organic and medicinal chemists [358,359,360]. Synthetic strategies significantly contribute to overcoming supply problems of marine peptides of interest, affording higher quantities of compounds required for large-scale biological assays. Marine cyclic peptides are also interesting models for molecular modifications and/or total synthesis for obtaining more potent compounds with improved properties. Total synthesis of marine natural peptides and analogues is very important for SAR studies, mechanism of action, and toxicity studies, when the amount of peptide extracted from marine organisms is very small and unprofitable [361]. As shown in Table 1, Table 2, Table 3, Table 4 and Table 5, many authors have explored the total or semi-synthesis of antimicrobial cyclic peptides and/or analogues, or performed structural modifications. In this section, the synthesis of some antimicrobial cyclic peptides that have been carried out on solid phase with resins and with the protective groups Fmoc and Boc are presented. Subsequently, other examples depict simple, green, and efficient strategies for synthesizing peptides.

**Scheme 1:**
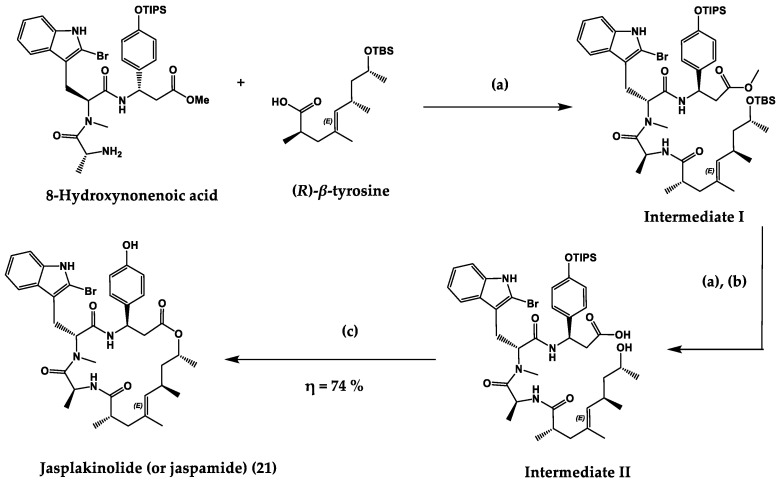
Synthesis of jasplakinolide (**21**). Reagents and conditions: (a) DCC, HOBT, THF, −20–0 °C, η = 80%; (b) Sol. aq. LiOH, then TIPSOTf 2,6-lutidine, followed sol. aq. K_2_CO_3_, η = 77%; (c) 2,4,6-Cl_3_-C_6_H_2_-COCl, DIPEA, then DMAP followed TBAF, THF, η = 74%.

As described in Table 1, jasplakinolide (**21**) exhibits important biological properties, including insecticidal, antifungal, and anthelminthic activities. To perform SAR studies, Ghosh A. and Moon D. [157] performed an enantioselective synthesis of **21** (Scheme 1) for further structural modifications. 

The coupling of the amino ester 8-hydroxynonenoic acid with (*R*)-*β*-tyrosine was performed in the presence of DCC and HOBT to obtain intermediate I. Subsequently, saponification was performed with aqueous LiOH to remove the *tert*-butyldimethylsilyl ether (TBS) group, affording the corresponding ester group in intermediate compound II. Then, the open product II was subjected to Yamaguchi macrolactonization protocol [362] with 2,4,6-trichlorobenzoyl chloride in the presence of DMAP. The removal of the TIPS group was carried out by treatment of the macrolactone with tetra-*n*-butylammonium fluoride (TBAF) yielding (+)-jasplakinolide (**21**) [157].

**Scheme 2:**
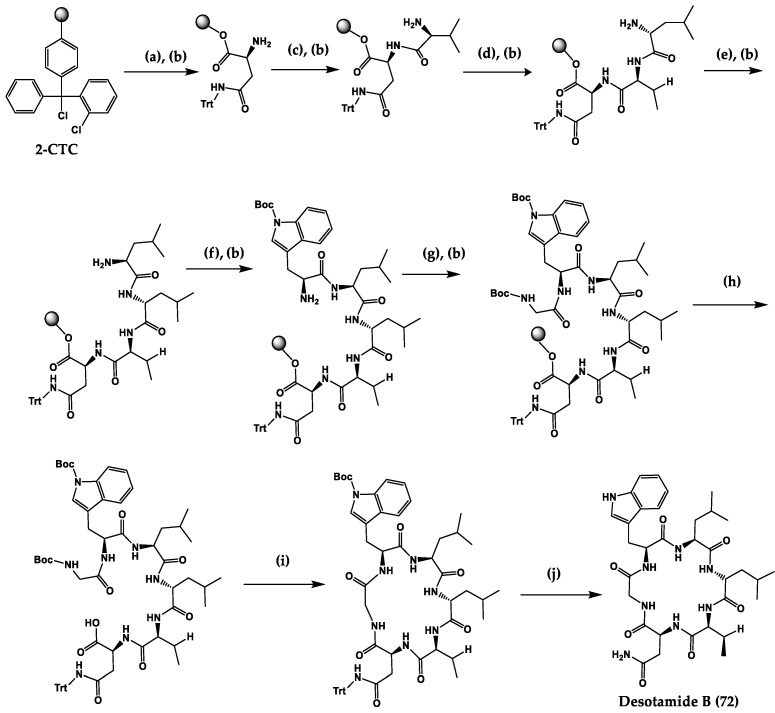
Solid-phase synthesis of desotamide B (**72**) on 2-CTC resin. Reagents and conditions: (a) *N*-*α*-(9-fluorenylmethoxycarbonyl)-*N*-γ-trityl-L-asparagine, DIPEA, DCM, 3 h; (b) 25% 4-methylpiperidine, DMF; (c) *N*-(9-fluorenylmethoxycarbonyl)-L-valine, DIC, HOBt, DMF/DCM 1:1, 4 h; (d) *N*-(9-fluorenylmethoxycarbonyl)-D-leucine, DIC, HOBt, DMF/DCM 1:1, 4 h; (e) *N*-(9-fluorenylmethoxycarbonyl)-L-leucine, DIC, HOBt, DMF/DCM 1:1, 4 h; (f) *N*-*α*-(9-fluorenylmethyloxycarbonyl)-*N*-in-*t*-butyloxycarbonyl-L-tryptophan, DIC, HOBt, DMF/DCM 1:1, 4 h; (g) *N*-*α*-(9-fluorenylmethyloxycarbonyl)-*N*-*ɤ*-*t*-butyloxycarbonyl-D-ornithine, DIC, HOBt, DMF/DCM 1:1, 4 h; (h) Hexafluoroisopropano/DCM 1:4, 30 min; (i) HBTU, DIPEA, DMF, 30 min; (j) TFA/TIPS/DCM, 50:5:45, 30 min, η = 37% (two last steps).

Tsutsumi L. et al. [210] developed the total synthesis of desotamide B (**72**) (Scheme 2) by using both SPPS and solution-phase macrolactamization. Through the fmoc solid-phase protecting strategy, the linear hexapeptide assembled on 2-chlorotrityl chloride resin (2-CTC) was used by the combination of DIC/HOBt coupling reagents; the cleavage of resin with a mixture of hexafluoroisopropanol/dichloromethane (DCM) made it possible to keep the side-chain-protecting groups intact. Recently, studies have reported that the solvent influences the macrocyclization of linear peptides, since its concentration often plays an important role in minimizing unwanted oligomers and polymer side products [363]. Therefore, it is generally recommended that the macrocyclization of linear peptides be performed under dilute submillimolar concentration [330]. Finally, global removal of all protecting groups using a combination of TFA/TIPS/DCM was performed to obtain the desired cyclic hexapeptide **72** [210]. 

**Scheme 3:**
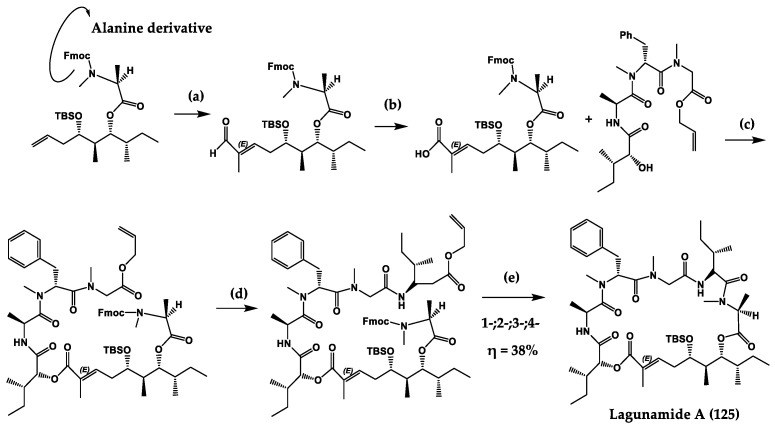
Synthesis of lagunamide A (**125**). Reagents and conditions: (a) Methacrylaldehyde, DCM, reflux, 87%, E:Z > 99:1; (b) NaClO_2_, NaH_2_PO_4_, *t*-ButOH, 2-methylbut-2-ene, r.t., 80%; (c) 2-Methyl-6-nitrobenzoic anhydride, DMAP, DCM, r.t., 56%; (d) (1) Et_2_NH/CH_3_CN, r.t., (2) *N*-(*tert*-butoxycarbonyl)-L-isoleucine, HATU, DIPEA, DCM, 0 °C—r.t., 91%; (e) (1) Pd(PPh_3_)_4_, PhNHMe, THF, r.t.; (2) Et_2_NH/CH_3_CN, rt; (3) HATU, DIPEA, DCM, r.t., (4) 40% aqueous HF, CH_3_CN, η = 38% (four last steps).

High efficacy for murine leukemia cell lines [364] and moderate antiswarming activity against *P. aeruginosa* [251] prompted Huang et al. [263] to develop a general synthetic approach for lagunamide A (**125**) (Scheme 3). The key feature in the synthesis included the preparation of four consecutive chiral centers at C_37__–__40_ and the final macrocyclization. The challenges in the synthesis of **125** were (i) macrolactonization [365] *versus* lactamization [366] for successful cyclization [336], (ii) proper protection of one ester group over another, and (iii) synthesis of the polyketide moiety [336]. The original strategy of using ring-closing metathesis for the macrocyclization did not work; thus, an alternative approach for ring closure was employed. The esterification to introduce the first amino acid unit and the aliphatic chain (first intermediate) resulted in significant epimerization. It is worth mentioning that the major product was the other isomer with epimerization at position *α* to the carbonyl group, suggested to occur after the formation of the ester bond. This problem was finally overcome by coupling the alcohol with the corresponding acyl chloride of the L-alanine derivative. The authors accomplished the synthesis of the revised structure for natural **125** starting from the first intermediates, such as the aliphatic chain, alanine, and isoleucine residues, and macrolactamization between the alanine and isoleucine moieties was performed to give **125**. 

**Scheme 4:**
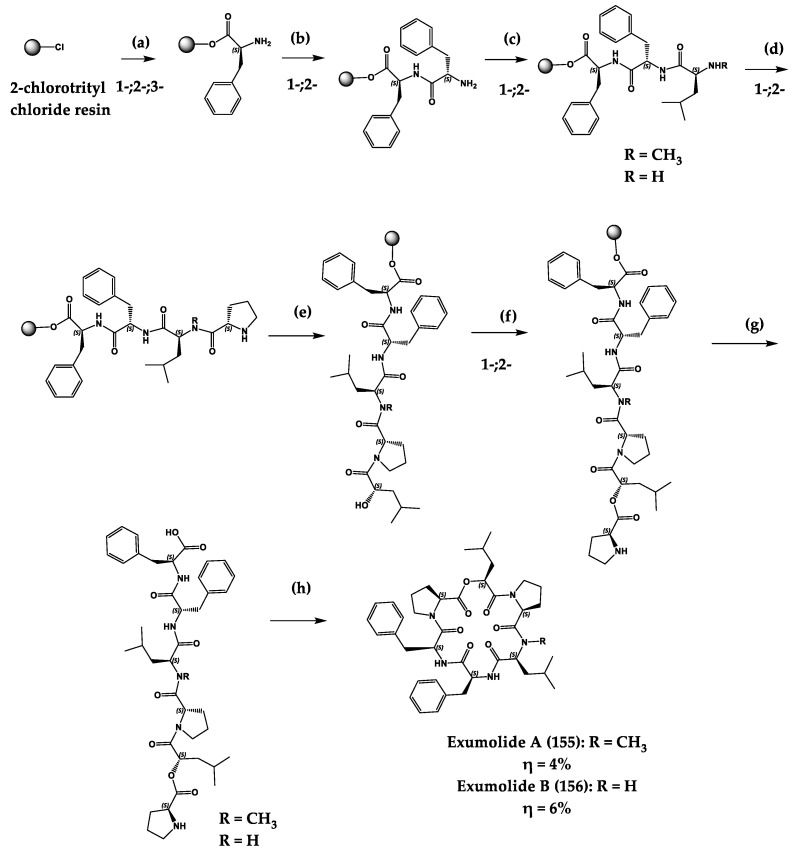
Synthesis route of exumolides A (**155**) and B (**156**). Reagents and conditions: (a) (1) *N*-(9-Fluorenylmethoxycarbonyl)-L-phenylalanine, DIPEA, DCM, 24 h, r.t.; (2) MeOH/DCM/DIPEA (3:7:1), (3) 20% piperidine in DMF; (b) (1) *N*-(9-fluorenylmethoxycarbonyl)-L-phenylalanine, HBTU, HOBt, DIPEA, DMF, 4 h, r.t. (2) 20% piperidine in DMF; (c) (1) *N*-*α*-fmoc-*N*-*α*-methyl-L-leucine/*N*-(9-fluorenylmethoxycarbonyl)-L-leucine, HBTU, HOBt, DIPEA, DMF, 4 h, r.t.; (2) 20% piperidine in DMF; (d) (1) *N*-(9-fluorenylmethoxycarbonyl)-L-proline, HATU, HOAt, DIPEA, DMF, 2 × 4 h, r.t., (2) 20% piperidine in DMF; (e) (*S*)-2-hydroxy-4-methylpentanoic acid, HATU, HOAt, DIPEA, DMF, 2 × 4 h, r.t.; (f) (1) *N*-(9-fluorenylmethoxycarbonyl)-L-proline, DIC, DMAP, DIPEA, DCM:DMF (95:5), 3 × 5 h, 45 °C, (2) 20% piperidine in DMF; (g) 20% TFA in DCM, 2 × 10 min, r.t.; (h) HATU, DIPEA, DCM, 48 h, r.t. Overall yield: η = 4% (**155**) and η = 6% (**156**).

To explore the biological properties of exumolides A (**155**) and B (**156**), Rahmadani, A. et al. [302] were interested in synthesizing peptides **155** and **156** (Scheme 4) using a combination of solid- and solution-phase methods. First, a linear precursor was synthesized using a solid-phase method on 2-CTC resin with the standard Fmoc strategy. The hydroxy acid, (*S*)-2-hydroxy-4-methylpentanoic acid, was prepared from its precursor L-leucine, and attached on the resin with a double-coupling protocol. The depside bond formation that was carried out at the end of the coupling process was particularly beneficial in the blockage of the diketopiperazine formation during Fmoc deprotection. In addition, Coin et al. [367] explained that the presence of a depside bond in the backbone would easily induce the formation of diketopiperazine. The coupling reaction involving *N*-methyl residue took advantage of HATU/HOAt through a double coupling protocol [368]. The use of (*S*)-2-hydroxy-4-methylpentanoic acid without the protecting group and the strategy of putting ester bond formation at the last step on the solid-phase successfully produced linear depsipeptides. The cyclic products **155** and **156** were obtained through HATU-based cyclization [302].

**Scheme 5:**
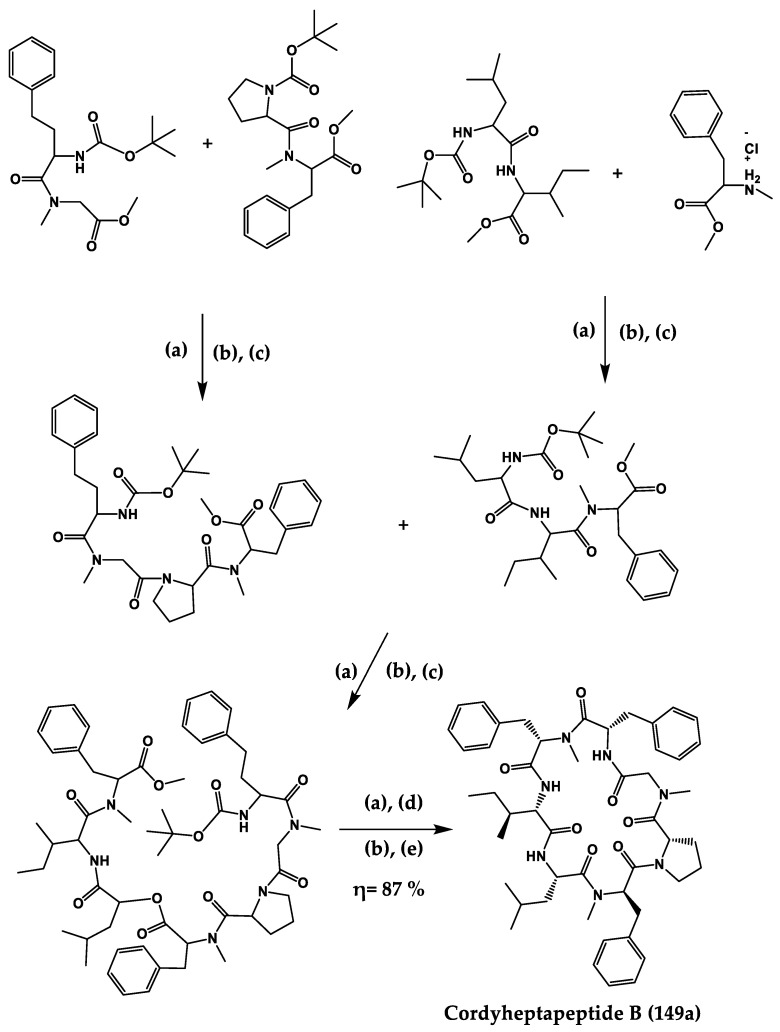
Synthetic pathway for cordyheptapeptide B (**149a**). Reagents and conditions: (a) LiOH, THF:H_2_O (1:1), r.t., 1 h; (b) TFA, chloroform, r.t., 1 h; (c) DIC, NMM, chloroform/DMF, r.t., 24 h; (d) DIC, pentafluorophenyl, r.t., 12 h; (e) TEA/NMM/pyridine, chloroform, 7 days, 0 °C, η = 87% (last step).

For investigation of biological activities, Rajiv Dahiya and Hemendra Gautam [293] synthesized an analogue of cordyheptapeptide A (**149**), the *N*-methylated cyclic peptide cordyheptapeptide B (**149a**) (Scheme 5), by coupling *N*-methylated tetrapeptide and tripeptide units. First, the deprotection at carboxyl and amino terminals, followed by cyclization of linear heptapeptide fragment was carried out. Required tetrapeptide and tripeptide units were prepared by coupling of Boc-protected dipeptides. Cyclization of the linear peptide unit was performed using the pentafluorophenyl ester method [369], which can be easily adapted to automated peptide synthesis systems [293,370]. 

As a final remark, it is important to highlight that, nowadays, green chemistry is an important concern and challenge in the synthesis of peptides, considering the urgency of protecting the environment from pollution, as well as ensuring that clean water and energy are available for future generations [371]. A high environmental impact can be observed due to the huge volume of solvents required for the peptide synthesis protocol by SPPS (such as Fmoc/*t*Bu) protection strategy [372], high amounts of reagents are also introduced to push the reaction to completion and minimize the formation of impurities [371]. In this sense, efforts to improve the environmental profile of the whole SPPS process and to obtain greener downstream purification processes are mandatory to ensure prioritizing of the quality and the purity of the crude peptides. Regarding cleavage steps, TFA still remains the most effective cleavage method in the SPPS Fmoc/*t*Bu protection strategy [373]. Nevertheless, a low amount of TFA (1–3%) mixed with an organic solvent is needed for cleavage of the side-chain-protected peptides from 2-CTC resins. Moreover, “green solvents” such as anisole and 1,3-dimethoxybenzene have been explored to replace DCM. The combination of coupling agents in the formation of peptide bonds in SPPS uses a methodology mainly based on DIC [374]. The benzotriazole family accounts for the first coupling reagent additives introduced for SPPS (HOBt, HOAt, and HBTU are prime examples), and these additives present an explosion hazard [375]. Moreover, they could induce skin sensitization after long-term exposure [376]. All derivatives introduced as autonomous coupling reagents, despite having greater stability, superior coupling efficiency, and lesser tendency to racemization, are also governed by the same classification (“Class 1 explosive category”) [377,378,379]. COMU belongs to the oxime family and shows higher coupling efficiency accompanied by lower epimerization and wide solubility in various solvents than other coupling agents [352,380].

## 4. Conclusions

Several antimicrobial cyclic peptides isolated from marine sources such as sponges, bacteria, cyanobacteria, fungi, and some invertebrates have been demonstrated to have significant antimicrobial activity against microorganisms as well as antibiotic-resistant microorganisms. Most of the cyclic peptides demonstrate major antibacterial and antifungal, followed by antiviral, activities, while a minor percentage demonstrate antiparasitic activity. Marine sponges appear to be among the most abundant reserves of marine natural products that are active against microorganisms. Numerous ecological explanations have shown that secondary metabolites produced by sponges often serve defensive purposes, protecting them from threats such as predator attacks, microbial infections, biofouling, and overgrowth by other sessile organisms [381,382]. The common characteristics of the cyclic peptides isolated from sponges include high degree of isomerism and similarities, as represented in the peptide families of koshikamides (**22**–**23**), microsclerodermins (**26**–**36**), mirabamides (**39**–**42**), papuamides (**51**–**52**), and theonellamide (**58**). Lithistid sponges are characterized by a high proportion of D and/or *N*-methylated amino acids.

Bacteria incorporate non-proteinogenic amino acids to prevent proteolysis of peptides through the stabilization of backbone conformation and/or by elimination of the enzyme recognition site [57]. Compounds derived from this source have non-amino acid moieties attached. It is also noteworthy that most cyclic peptides from bacteria demonstrate antifungal and antiviral activities. Cyanobacteria, fungi, and other marine invertebrates have smaller peptides compared to sponges.

Most cyclic peptides from marine organisms, especially sponges, possess proline-rich cyclic peptides, an interesting class of peptides with a wide range of biological functions. The proline residue in these molecules plays an important structural role, reducing the conformational flexibility, leading to the maintenance of a rigid structure which, in turn, leads to improved bioactivity. In addition, the presence of *N*-containing heterocycles can be observed, which make a significant contribution to antimicrobial activity. Furthermore, the diverse structures of isolated cyclic peptides range from glyco-, lipo-, and depsi-, with large bicyclic rings.

Some cyclic peptides, including nocathiacins I–III (**91**–**93**), unnarmicins A (**114**) and **C** (**115**), sclerotides A (**160**) and B (**161**), and plitidepsin (**174**), can be highlighted as not only demonstrating high potency in vitro, but also promising in vivo results. 

For drug discovery and development, large quantities of material are required for large-scale biological assays. Synthetic strategies contribute significantly to overcoming supply problems of marine peptides of interest. Solid phase using 2-CTC resin through the combination of solution-phase synthesis is the most used technique for synthesizing cyclic peptides. Protecting groups, such as Fmoc-, Boc-, methyl, TBS and TIPS, demonstrated to be the major synthetic strategies to prevent undesired reactions and to achieve chemoselectivity in a subsequent chemical reaction. Marine cyclic peptides are promising drug candidates, exhibiting very interesting biological properties. Their biological properties and structural features have attracted attention with respect to total synthesis and structure modification, which still present several challenges. This review summarizes several promising marine cyclic peptides with relevant antimicrobial activity. Furthermore, this comprehensive compilation is extremely valuable and interesting for understanidng the research strategies and recent progress in the marine cyclic peptide field, as well as inspiring and guiding microbiologists and medicinal chemists in the discovery of new antimicrobial drug candidates from marine sources.

## Data Availability

Not applicable.
